# ROS‐Driven Nanoventilator for MRSA‐Induced Acute Lung Injury Treatment via In Situ Oxygen Supply, Anti‐Inflammation and Immunomodulation

**DOI:** 10.1002/advs.202406060

**Published:** 2025-03-19

**Authors:** Zheng Luo, Qi Wang, Xiaotong Fan, Xue Qi Koh, Xian Jun Loh, Caisheng Wu, Zibiao Li, Yun‐Long Wu

**Affiliations:** ^1^ State Key Laboratory of Cellular Stress Biology Fujian Provincial Key Laboratory of Innovative Drug Target Research School of Pharmaceutical Sciences Xiamen University Xiamen 361102 China; ^2^ Institute of Materials Research and Engineering (IMRE) Agency for Science Technology and Research (A*STAR) 2 Fusionopolis Way, Innovis #08‐03 Singapore 138634 Republic of Singapore; ^3^ Institute of Sustainability for Chemicals Energy and Environment (ISCE2) Agency for Science, Technology and Research (A*STAR) 1 Pesek Road, Jurong Island Singapore 627833 Republic of Singapore; ^4^ Department of Materials Science and Engineering National University of Singapore Singapore 117576 Republic of Singapore

**Keywords:** acute lung injury, anti‐inflammatory, enzyme therapy, oxygen supply, ROS clearance

## Abstract

Hypoxia, inflammatory response and pathogen (bacterial or viral) infection are the three main factors that lead to death in patients with acute lung injury (ALI). Among them, hypoxia activates the expression of HIF‐1α, further exacerbating the production of ROS and inflammatory response. Currently, anti‐inflammatory and pathogen elimination treatment strategies have effectively alleviated infectious pneumonia, but improving lung hypoxia still faces challenges. Here, a vancomycin‐loaded nanoventilator (SCVN) containing superoxide dismutase (SOD) and catalase (CAT) is developed, which is prepared by encapsulating SOD, CAT and vancomycin into a nanocapsule by in situ polymerization. This nanocapsule can effectively improve the stability and loading rate of enzymes, and enhance their enzyme cascade efficiency, thereby efficiently consuming •O_2_^−^ and H_2_O_2_ to generate O_2_ in situ and reducing ROS level. More interestingly, in situ O_2_ supply can effectively relieve lung hypoxia to reduce HIF‐1α expression and balance the number of M1/M2 macrophages to reduce the levels of TNF‐α, IL‐1β and IL‐6, thereby alleviating the inflammatory response. Meanwhile, vancomycin can target and kill MRSA, fundamentally solving the cause of pneumonia. This nanoventilator with antibacterial, anti‐inflammatory, ROS scavenging and in situ O_2_ supply functions will provide a universal clinical treatment strategy for ALI caused by pathogens.

## Graphical abstract

A ROS‐driven in situ oxygen supply nanoventilator based on enzyme nanocapsule can convert ROS into O_2_ through enzyme cascade reaction between SOD and CAT to relieve lung hypoxia, and kill MRSA by releasing vancomycin, which effectively reduces HIF‐1α expression and balances pro‐inflammatory/anti‐inflammatory macrophages ratio, thereby reducing the levels of ROS and pro‐inflammatory cytokines, effectively treating MRSA‐induced acute lung injury.

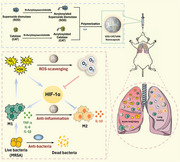

## 1 Introduction

Lungs are the most critical organs in the human respiratory system that perform gas exchange through inhaled air to obtain oxygen necessary for cellular respiration.^[^ ^1^ ^]^ However, they are susceptible to infection by potentially pathogenic microorganisms (such as bacteria, viruses, and fungi) due to prolonged exposure to the air, resulting in severe acute lung injury (ALI), such as COVID‐19 and *Staphylococcus aureus* pneumonia.^[^ ^2^ ^]^ ALI usually causes rapid recruitment and stimulation of pulmonary inflammatory cells like pro‐inflammatory (M1) macrophages and neutrophils, accompanied by inflammatory cytokine storm and high reactive oxygen species (ROS) levels, which will damage lung cells such as alveolar epithelium and pulmonary capillary endothelial cells, and lead to pulmonary edema and refractory hypoxemia, and respiratory failure in severe cases, thus threatening the lives of individuals.^[^ ^3^ ^]^ In addition, hypoxia in the lungs will cause the upregulation of hypoxia‐inducible factor‐1α (HIF‐1α) that is widely expressed in immune cells like M1 macrophages, promoting the massive release of proinflammatory cytokines, further exacerbating the pulmonary inflammatory response.^[^ ^4^ ^]^ Currently, the treatment of acute pneumonia has made significant progress in anti‐inflammatory, removing ROS and pathogens,^[^ ^5^ ^]^ but it is rare to pay attention to the effective delivery of oxygen.

Oxygen plays an indispensable part in cellular energy metabolism, which is essential for cell growth, development, and survival.^[^ ^6^ ^]^ Currently, oxygen therapy (such as hyperbaric oxygen therapy) is a common clinical treatment for relieving pulmonary hypoxia.^[^ ^7^ ^]^ However, the commonly used form in clinical practice is gaseous oxygen, which has issues such as poor tissue permeability and low transport efficiency, necessitating higher oxygen delivery concentrations that may also cause oxidative damage to the lungs.^[^ ^8^ ^]^ How to efficiently deliver oxygen while reducing ROS and inflammatory responses is crucial for the treatment of acute pneumonia. In recent years, in situ oxygen delivery systems have shown high oxygen delivery efficiency in wound healing, tumor treatment, etc., effectively improving the hypoxic environment of tissues and enhancing therapeutic effects.^[^ ^9^ ^]^ Most of them achieve local oxygen supply by converting H_2_O_2_ into O_2_. Inspired by this, developing an oxygen supply system with highly toxic free radicals as the oxygen source can kill two birds with one stone for the treatment of acute pneumonia. Bacterial‐induced acute lung injury usually leads to the generation of a large amount of ROS in local lung tissue, especially H_2_O_2_ and •O_2_^−^. Catalase (CAT) and superoxide dismutase (SOD) are natural antioxidant systems of cells. The former can catalyze the conversion of •O_2_^−^ into H_2_O_2_ and O_2_, while the latter can convert the H_2_O_2_ produced by the former into O_2_, thereby efficiently converting toxic ROS into O_2_ through enzyme cascade reactions,^[^ ^10^ ^]^ improving the hypoxic environment and exerting a good anti‐inflammatory effect. However, due to the large molecular weight and poor stability of natural enzymes, they have problems such as low loading efficiency and difficult delivery, which seriously limit their clinical application. In the early stage, our research group developed a simple and efficient natural enzyme delivery system,^[^ ^11^ ^]^ which can realize the co‐loading of multiple enzymes at the same time, effectively increase the enzyme stability and loading capacity, and improve the enzyme cascade efficiency through the nanosized effect.

**Scheme 1:**
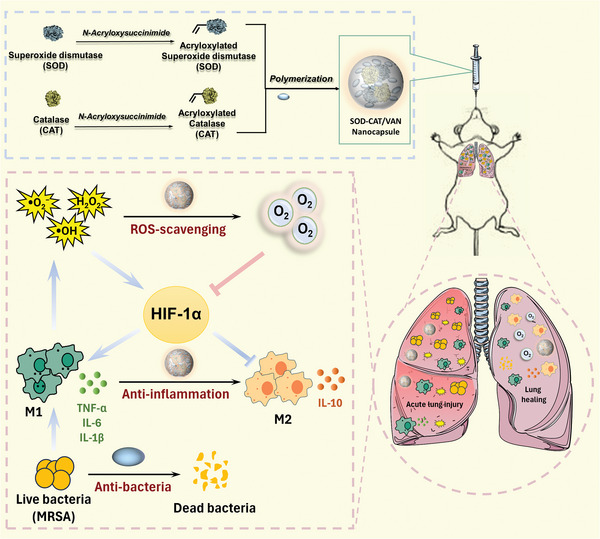
Schematic illustration of the design of SCVN with ROS removal, in situ oxygen supply, antibacterial and anti‐inflammatory properties to cure acute lung injury.

Due to the drug resistance and high virulence of Methicillin‐resistant *Staphylococcus aureus* (MRSA), the acute lung injury caused by MRSA infection has a stronger inflammatory response, resulting in severe tissue damage and more severe clinical symptoms.^[^ ^12^ ^]^ Vancomycin is a glycopeptide antibiotic that effectively inhibits the cell wall synthesis of MRSA without being affected by the common resistance mechanisms of MRSA (such as β‐lactamase and altered penicillin‐binding protein). It has a strong antibacterial effect on MRSA and is one of the clinical first‐line drugs for the treatment of MRSA infection.^[^ ^13^ ^]^ Herein, we reported an injectable nanoventilator based on enzyme nanocapsule loaded with SOD and CAT and vancomycin for the treatment of ALI caused by MRSA, with the ability to clear ROS, produce oxygen, reduce inflammatory cytokine levels, and facilitate macrophages shifting from pro‐inflammation M1‐like phenotypes to anti‐inflammation M2 phenotypes, as shown in **Scheme** **1**. The SOD‐CAT/Vancomycin nanocapsule (SCVN) was prepared by in situ polymerization with acryloxylated SOD and CAT, acrylamide (Acryl), N, N’‐methylene bisacrylamide and antibacterial monomers vancomycin. This design can not only shield the enzyme from external interference by forming nanocapsules, but also improve enzyme stability and enzyme loading. Nanocapsules can also enhance enzyme cascade reaction efficiency by shortening the intermediate distance between SOD and CAT. In addition, SCVN can use ROS (H_2_O_2_ and •O_2_^−^) as a substrate to generate O_2_ in situ, thereby improving the hypoxic environment of the lungs, reducing the expression of HIF‐1α, which could promote the polarization of macrophages toward M2 phenotype to balance the number of M1/M2 macrophages, alleviating the inflammatory response. This nanoventilator based on enzyme nanocapsule possesses multiple functions, such as ROS removal, anti‐inflammation, antibacterial, and sustained in situ oxygen supply, providing a new therapeutic strategy for MRSA‐induced acute lung tissue injury.

## 2 Results and Discussion

### 2.1 Preparation and Characterization of Multifunctional SOD‐CAT/Vancomycin Nanocapsules (SCVN)

**Figure 1:**
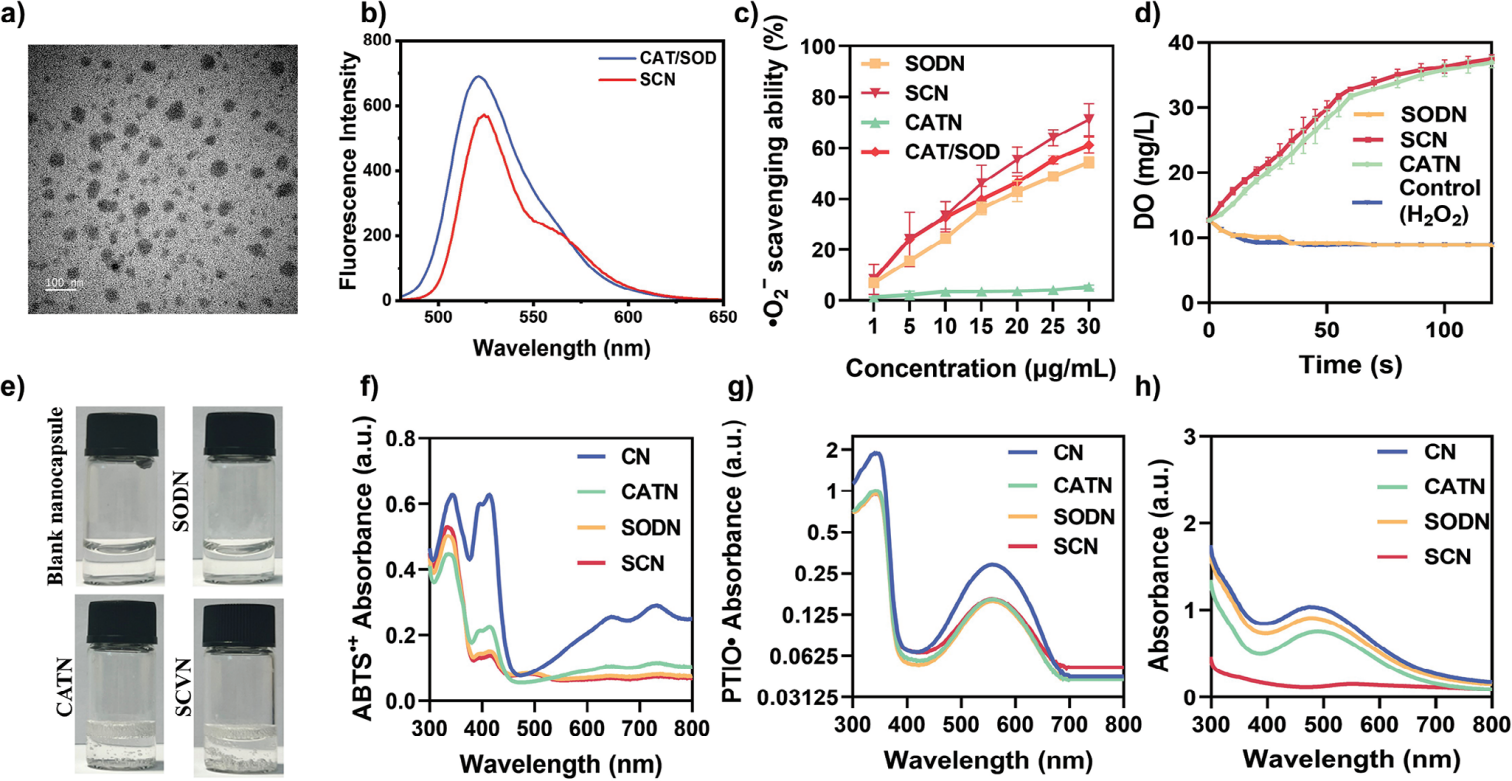
In vitro characterization of prepared nanocapsule. a) TEM image of SCVN, scale bar:100 nm. b) Fluorescence spectra of Cy3‐CAT and FITC‐SOD mixture, and the nanocapsule constructed from Cy3‐CAT and FITC‐SOD excited at 480 nm. c) Scavenging ability of different nanocapsules to •O_2_^−^. d) Dissolved oxygen levels were treated with different nanocapsules and hydrogen peroxide (H_2_O_2_) to evaluate the CAT activity. e) Images of different nanocapsules (1 mg mL^−1^) catalyzing H_2_O_2_ to produce oxygen in 0.3% H_2_O_2_ solution. Scavenging abilities of f) ABTS^•+^, g) PTIO•, and h) •OH at different nanocapsules treated with the concentration of 50 µg mL^−1^.

The SCVN nanogels were designed through in situ polymerization as demonstrated in Scheme 1. The morphology and size of the prepared SOD‐CAT/Vancomycin nanocapsules (SCVN) were investigated via transmission electron microscopy (TEM). From **Figure** **1**a, the SCVN is spherical with a size of ～50 nm. DLS analysis (Figure S1a, Supporting Information) showed that the size of SCVN was ～56.2 nm and its zeta potential (Figure S1b, Supporting Information) was ～−21.7 mV. To further confirm whether the nanocapsule can effectively encapsulate SOD and CAT, we labeled CAT and SOD with Cy3 and FITC probes, respectively. As shown in Figure 1b, under excitation with a 480 nm laser, SOD‐CAT nanocapsules (SCN) exhibit fluorescence resonance energy transfer (FRET), indicating that the spatial distance between SOD and CAT is very close. This suggests that both enzymes can be simultaneously encapsulated within the nanocapsule. In addition, we used Cy3.5, rhodamine B and FITC to label SOD, CAT and vancomycin respectively, and encapsulated them into the nanocapsule by in situ polymerization and observed their morphology by confocal microscopy. As shown in Figure S2 (Supporting Information), these three fluorescence signals exhibit a good overlap effect, indicating that SOD, CAT, and vancomycin can be effectively encapsulated within the same nanocapsule. Moreover, FTIR spectrums of the obtained nanocapsules are shown in Figure S3 (Supporting Information), characteristic peak at ～1240 cm^−1^ representing the vibration of ether bond which existed in the vancomycin and SCVN group without in the SCN group verifying the synthesis of SCVN nanocapsule. Besides, the peaks (similar to SCN) at ～1400 cm^−1^ in SCVN are redshifted by the introduction of vancomycin.

We also examined the enzyme activity before and after loading enzymes and vancomycin. The test results indicated the polymerization and loading vancomycin had a limited effect on the enzyme activity. Figure S4 (Supporting Information) showed the catalytic activity of SCVN kept 84.5% of native CAT activity, CAT nanocapsule (CATN) kept 91.6% of the native CAT activity. The SOD activity of native SOD, SOD nanocapsule (SODN), SCVN showed a measured IC50 of 4.18 , 4.74 and 4.42 nM, respectively (Figure S5, Supporting Information). This indicated the catalytic activity of SODN kept 88.2% of native SOD activity, SCVN kept 94.5% of the native SOD activity. In addition, we simulated the release behavior of vancomycin and enzymes in the lung microenvironment at pH 6.8. As shown in Figure S6 (Supporting Information), the enzyme is hardly released in the lung microenvironment, which may be since the nanocapsules formed by the in situ polymerization method can tightly encapsulate the enzyme. The release of vancomycin within 24 h is almost 100%, which may be because vancomycin is directly encapsulated and does not participate in the polymerization reaction. Interestingly, by comparing the loss of CAT enzyme activity before and after the formation of nanocapsules in a 60 °C water bath, it can be found that this nanocapsule structure can effectively improve the stability of the enzyme. The enzyme activity of CATN decreased by ～5% after incubation in a 60 °C water bath for 30 min (Figure S7, Supporting Information), while the enzyme activity of CAT decreased by ～35%.

### 2.2 Evaluation of In Vitro Cascade Catalytic Activity of SCN

After the successful preparation and characterization, it is essential to verify the cascade catalytic activity of the SCN system. Applying a SOD assay kit to evaluate the SOD activity and explore any changes in enzyme activities after the loading of the prepared nanocapsule with SOD. Briefly, xanthine and xanthine oxidase (XOD) produce the •O_2_^−^ which undergoes a reduction reaction with WST‐8 to form a water‐soluble formaldehyde dye absorbing at 560 nm. As anticipated, SOD can inhibit the reduction of WST‐8 and the formation of formazan, concluding that the darker color of the formazan represents less activity of SOD. As shown in Figure 1c, the •O_2_^−^‐scavenging rate was measured at various concentrations of different nanocapsules. The •O_2_^−^ scavenging capacity of the SOD nanocapsule (SODN) progressively increased with concentration. From the concentration of 15 µg mL^−1^, the apparent activity of SOD in SCN was higher than in the other groups. By increasing the concentration of SCN to 30 µg mL^−1^, the rate of removal of •O_2_^−^ was up to 71%, compared with SODN (～54%) and the mixture of CAT and SOD (～61%).

The CAT capacity was investigated by recording the dissolved oxygen concentration by a portable dissolved oxygen tester after the addition of H_2_O_2_. To observe the oxygen generation precisely, we firstly normalized the oxygen concentration in the atmosphere at the initial. Figure 1e displayed an abundance of bubbles have emerged when 0.3% H_2_O_2_ reacted with the CATN and SCN, and the contents of dissolved oxygen in the mixture augmented instantly from 8.7 up to 25.8 and 32 mg L^−1^ within 50 s, respectively (Figure 1d). However, the dissolved oxygen levels of other groups without CAT remained essentially unaltered (maintained at 8.7 mg L^−1^) suggesting the catalytic performance of CAT reacting with H_2_O_2_ and the stability of both enzymes by polymerization.

### 2.3 ABTS^•+^, PTIO• and •OH Scavenging Ability of SCN

Overproduction of ROS in injury sites can cause fibrotic scarring, DNA damage, cellular senescence, and worsened inflammation, all of which can seriously impede the healing process.^[^ ^14^ ^]^ Therefore, the management of ALI needs to create a substance that effectively scavenges ROS, preserves the equilibrium of the antioxidant defense system, and lowers inflammation. Therefore, we next examined the ability of SCN to scavenge ROS from ABTS**^•+^,** PTIO•, and •OH.

K_2_S_2_O_8_ could oxidize ABTS to a stable radical (ABTS**^•+^**) which is the blue solution with a distinctive absorbance at 734 nm, and it could be utilized for tracking color fading when an electron donor diminishes it. As shown in Figure 1f, SCN possessed the better ABTS^•+^ scavenging effect with a weaker absorbance at 734 nm compared with other composites. Similarly, the blue color of the ABTS**^•+^** solution became lighter after adding SCN (Figure S8, Supporting Information), demonstrating SCN has superior ABTS^•+^ scavenging ability.

PTIO•, one of the oxygen free radicals, has an absorbance peak at 557 nm and a purple coloration in solution. SCN possessed better PTIO• scavenging capacity with the comparison of other composites, with a lower absorbance peak intensity at 557 nm (Figure 1g) and lighter purple color in solution (Figure S8, Supporting Information).

Since •OH is a frequent ROS oxidization production that may negatively impact a variety of biomolecules in vivo, the preparation possessing the ability to eliminate •OH would offer noteworthy advantages in limiting an overabundance of ROS. It is known that salicylate could detect •OH and its solution has a distinctive absorbance at 510 nm. Figure 1h indicated that in the full‐wavelength scan, the SCN exhibited a lower absorbance at 510 nm than the control group. Similar results are also shown in Figure S8 (Supporting Information), showing that the less purple, the stronger the •OH removal ability.

In summary, the consequence of the free radical (such as ABTS**^•+^**, PTIO•, and •OH) ‐scavenging assay showed the in vitro ROS elimination capacities of SCN, which manifested their superiority and potential for antioxidant therapy.

### 2.4 Intracellular ROS Depletion and O_2_ Generation Ability of SCN

**Figure 2:**
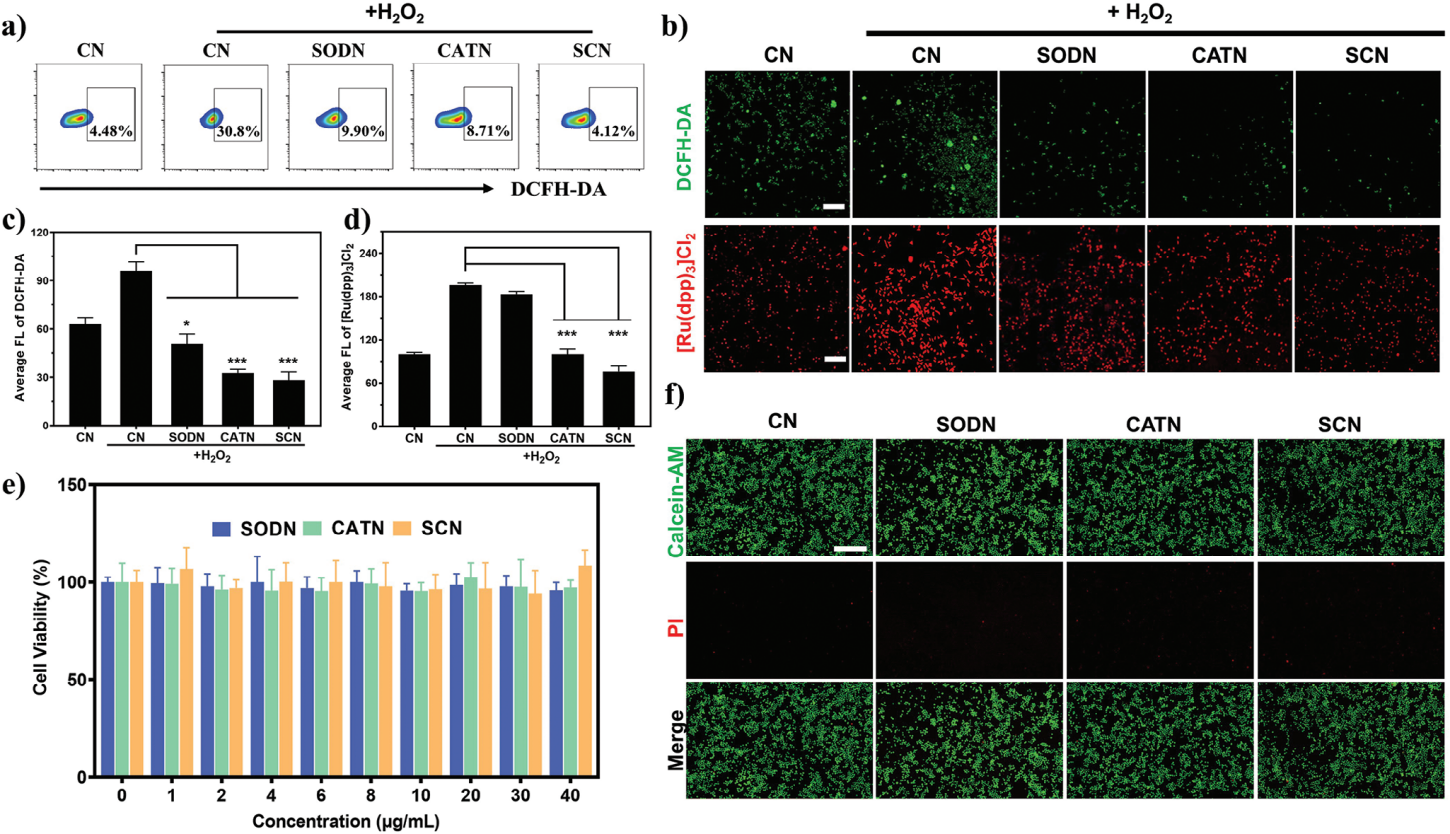
Evaluation of the antioxidation and oxygen generation of SCN. a) Fluorescence intensity of intracellular ROS measured by flow cytometry. b) Representative fluorescence images of intracellular ROS detected by DCFH‐DA probe and O_2_ generation assessed by O_2_ probe [Ru(dpp)_3_]Cl_2_. Scale bar: 200 µm. The quantitative studies of c) ROS depletion and d) O_2_ generation in RAW 264.7 cells using ImageJ software (*n* = 3). e) Cell viability of RAW 264.7 cells with various treatments after 24 h. f) Live/dead assay of RAW 264.7 cells cultured for 24 h under different conditions. Scale bar: 100 µm. Statistical comparisons were analyzed by One‐way ANOVA Tukey's multiple comparisons tests. *p < 0.05, **p < 0.01, ***p < 0.001, ****p < 0.0001 vs H_2_O_2_ group.

The prepared nanocapsules showed excellent SOD and CAT activities in vitro, which is worthy of further exploration in intracellular antioxidant application. We anticipated that SCN can alleviate this oxidative environment because CAT enzyme activity can consume H_2_O_2_ and generate O_2_. DCFH‐DA was used to determine ROS levels in RAW 264.7 cells treated with SCN to assess the oxidative stress damage of H_2_O_2_. As shown in **Figure** **2**a and Figure S9 (Supporting Information), the flow cytometry results demonstrated ROS in the SCN group ～7.5 times lower than the control group. Similarly, according to fluorescence microscopy (Figure 2b), the SCN group has lower green fluorescence intensity, indicating that it could attenuate the impact of H_2_O_2_ and has a better ROS scavenging capacity.

In addition, the O_2_ level in RAW 264.7 cells was measured with an oxygen probe ([Ru(dpp)_3_]Cl_2_). Therefore, the lighter red fluorescence intensity of [Ru(dpp)_3_]Cl_2_ means more O_2_ generation. As shown in Figure 2b, the red fluorescence intensity in RAW 264.7 cells treated with SCN was significantly weaker compared with the control group. Furthermore, these fluorescence quantitative results (Figure 2c‐d) also indicated the strong ROS scavenging and O_2_ generation ability of SCN.

Since the biosafety of the nanocapsule is critical for the treatment of ALI, we investigated its cytotoxicity through MTT assay. Figure 2e showed that the prepared SCN was almost not cytotoxic and could promote RAW 264.7 cell viability even at low concentrations, and it also showed little toxicity to human embryonic lung fibroblasts (MRC‐5) (Figure S10, Supporting Information), indicating that SCN had no detrimental effect on cell health. Meanwhile, we used a live/dead staining kit to detect the viability of RAW 264.7 cells after treatment with different nanocapsules (Figure 2f), further verifying that the prepared nanocapsules had good biosafety.

In summary, SCN with a nontoxic nature could remove ROS and generate oxygen to mitigate the adverse impacts of oxidative stress damage on cellular survival.

### 2.5 Anti‐Inflammatory Effect of SCN

**Figure 3:**
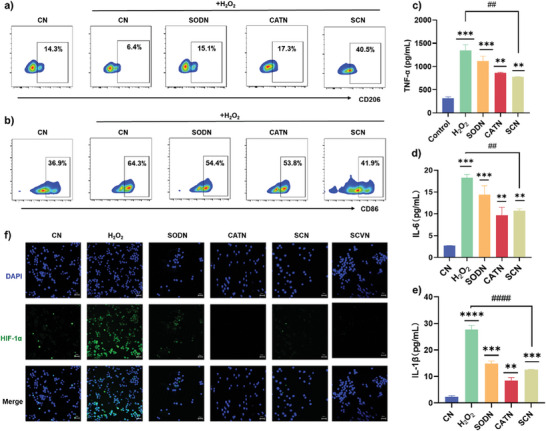
Effect of SCN on anti‐inflammation and polarization of macrophages. a) M2‐associated marker (CD206) and b) M1‐biomarker (CD86) expression after different treatments analysed by flow cytometry. H_2_O_2_‐treated macrophages served as the positive control (*n* = 3). Evaluation of the inflammation cytokines c) TNF‐α, d) IL‐6, and e) IL‐1β levels of the RAW 264.7 cells after various treatments using ELISA kits. f) Immunofluorescence images of HIF‐1α expression of RAW 264.7 cells treated with different conditions during 24 h. Scale bar: 50 µm. The means ± SD of the data from three separate experiments are shown. Statistical comparisons were analyzed by One‐way ANOVA Tukey's multiple comparisons tests. *p < 0.05, **p < 0.01, ***p < 0.001, ****p < 0.0001 vs control group. ^#^ *p* < 0.05, ^##^ *p* < 0.01, ^###^ *p* < 0.001, ^####^ *p* < 0.0001 vs H_2_O_2_ group.

Macrophages are recognized as critical cells in the immunological response to microbial infection or oxidate damage, playing an important role in inflammation induced by numerous diseases.^[^ ^15^ ^]^ Previous studies demonstrated that natural antioxidant enzymes can polarize pro‐inflammatory M1‐like macrophages to anti‐inflammatory M2 phenotypic.^[^ ^16^ ^]^ Therefore, we explored whether the SCN could induce the polarization of RAW 264.7 cells by examining some surface markers such as CD206 and Arg‐1 (M2 biomarkers), CD86 and iNOS (M1 biomarkers) through flow cytometry, qPCR, and immunofluorescence staining methods. Flow cytometry results (**Figure** **3**a–b; Figure S11, Supporting Information) showed that compared with H_2_O_2_‐induced RAW 264.7 cells, SODN, CATN, and SCN treated group could significantly decrease the expression of M1 marker CD86, while the levels of anti‐inflammatory M2 marker CD206 increased. Thus, SCN can successfully induce RAW 264.7 cells to polarize from the M1 phenotype to the tissue repair M2 phenotype, thereby well balancing the number of M1/M2 macrophages to improve the inflammatory response. Meanwhile, we further perform phenotypic analysis of macrophages by evaluating the change of M1 marker iNOS and M2 marker Arg‐1. Specifically, As shown in Figure S12 (Supporting Information), SCN significantly decreased the mRNA expression of iNOS in RAW 264.7 cells while SCN could promote the gene expression of Arg‐1. Figures S13 and S14 (Supporting Information) also indicated that the SCN‐treated RAW 264.7 cells demonstrated less green fluorescence and more red fluorescence. Therefore, the qPCR and immunofluorescence staining results showed consistency with the flow cytometry, indicating SCN could induce macrophage polarization toward anti‐inflammatory M2 macrophages, thereby effectively balancing the ratio of M1/M2 macrophages. When stimulated by H_2_O_2_, macrophages produce large amounts of inflammation cytokines like TNF‐α, IL‐6 and IL‐1β, which are positively associated with disease development and the presentation of acute inflammation. Therefore, we studied the levels of proinflammatory cytokines to investigate the anti‐inflammation effect of SCN. As shown in Figure 3c–e, SCN significantly reduced TNF‐α, IL‐6 and IL‐1β levels in H_2_O_2_‐induced RAW 264.7 cells. Furthermore, for the purpose of exploring the anti‐inflammatory pathway and mechanism of SCN, we first investigated whether SCN could affect hypoxia‐inducible factor‐1α (HIF‐1α) expression, the overexpression of which would aggravate oxidative stress and inflammatory response. Under hypoxic conditions, this factor would be activated in immune cells such as macrophages, but its expression was almost undetectable under normal conditions. Therefore, to explore the effect of SCVN on HIF‐1α expression through continuous oxygen supply, we constructed a RAW 264.7 cell model pretreated with H_2_O_2_ under hypoxia for evaluation. From the immunofluorescence images of Figure 3f, we observed less green fluorescence intensity in nanocapsules‐treated groups, indicating that the addition of CAT and SOD suppressed the expression of HIF‐1α through the generation of oxygen (Figure 1e and Figure 2b). Next, the protein expression of HIF‐1α was detected by Western blotting. The results showed that compared with the H_2_O_2_ group, SCVN treatment reduced protein expression levels of HIF‐1α confirming the alleviation in hypoxia and inflammation after SCVN treatment (Figure S15, Supporting Information). HIF‐1α plays an important role in inflammation, so SCVN can suppress the HIF‐1α signaling pathway to display its anti‐inflammatory activity by relieving hypoxia conditions, thereby enabling macrophages to shift from M1 phenotype to M2 phenotype, showing great clinical potential for the treatment and prevention of ALI.

### 2.6 Antibacterial Activity of SCVN

**Figure 4:**
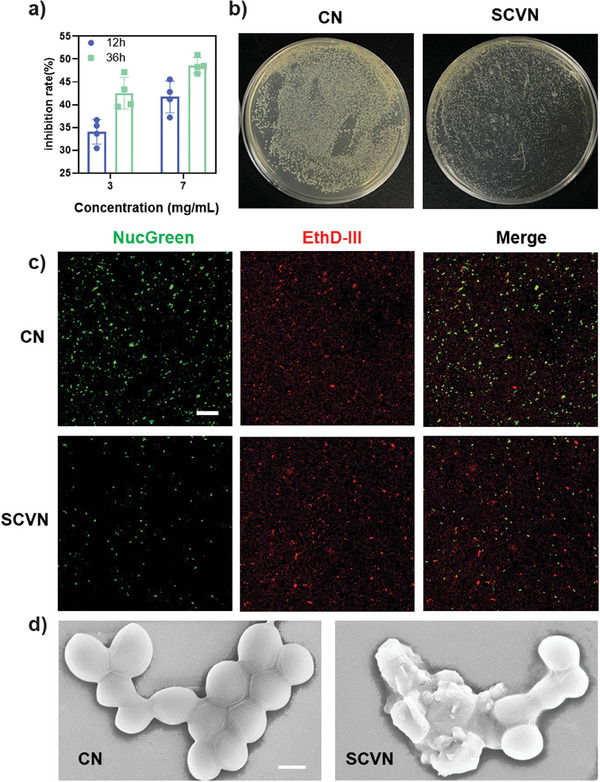
Examination of the antibacterial capacity of SCVN. a) Quantitative assessment of MRSA suppression by SCVN. b) Typical images of optical bacterium clones treated differently on an LB agar plate (left: control; right: SCVN). c) Typical fluorescence images by various treatments against MRSA using live/dead tests. All the scale bars are 100 µm. d) SEM image of MRSA without and with SCVN. Scale bar: 300 nm. (Error bars represent mean ± s.d.).

MRSA infection is considered another obstacle in the treatment process of ALIcaused by bacterial infection.^[^ ^17^ ^]^ Therefore, the antibacterial properties of SCVN of the vancomycin were evaluated in vitro. Since vancomycin has a good effect on gram‐positive bacteria, we took MRSA as the model for the antibacterial experiment. As shown in **Figure** **4**a and Figure S16 (Supporting Information), with the increase of SCVN concentration, the antibacterial effect was enhanced, and the antibacterial rate could reach ～50%. In addition, the antimicrobial visual image results were aligned with the previous results (Figure 4b). It is evident that the control group exhibits a high degree of colony formation but there is less colony formation in the SCVN group. To further validate the antibacterial effect of SCVN, we evaluated the activity against MRSA using a live/dead staining kit. The results (Figure 4c) confirmed that the number of bacteria treated with SCVN was less than that of the untreated group, as shown by a significant reduction in green fluorescence intensity. Through quantitative analysis of fluorescence intensity, the ratio of green fluorescence intensity to red fluorescence in the MRSA group treated with SCVN is significantly lower than that in the untreated group (Figure S17, Supporting Information), indicating that it has a good antibacterial effect. Besides, we further examine the antibacterial activity of SCVN using SEM characterization. As shown in Figure 4d, compared with untreated MRSA, the cell wall of MRSA treated with SCVN became obviously rough, wrinkled, and even damaged, which once again demonstrated its good antibacterial effect. In summary, the SCVN has good antibacterial properties, which can provide a possibility for the treatment of ALI.

### 2.7 SCVN Alleviating MRSA‐Induced ALI

**Figure 5:**
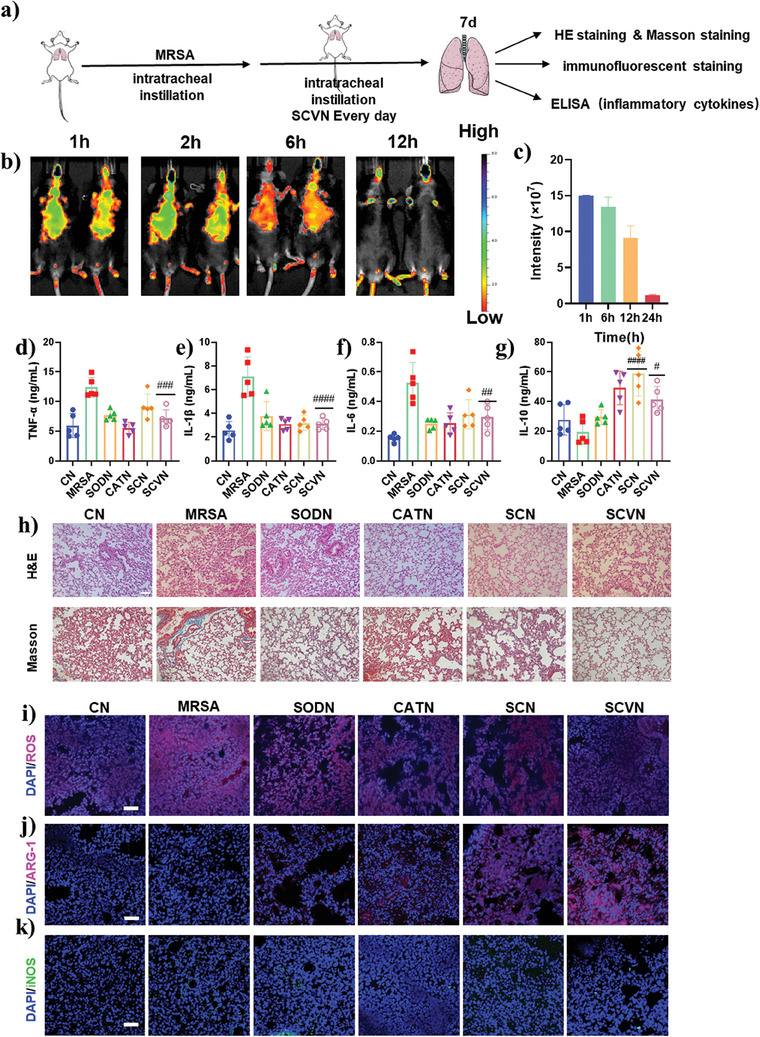
The distribution and curative strategy of SCVN in ALI mice. a) Schematic diagram to display the experimental arrangement. b) The in vivo biodistribution of SCVN after different time points (1, 6, 12, and 24 h). and c) the corresponding fluorescence intensity statistics. The doses of SCVN‐rhodamine B for bioimaging were 5 mg kg^−1^. Analysis of inflammatory cytokines such as d) TNF‐α, e) IL‐ 1β f) IL‐6, and g) IL‐10 of the lung after different treatments using ELISA kits. h) The lung tissues of the healthy mice and the ALI animals treated with PBS, SODN, CATN, SCN, and SCVN were stained with H&E and Masson staining. Scale bar: 50 µm. Fluorescence images of i) ROS, j) Arg‐1, and k) iNOS stained lung slices. Scale bar: 50 µm. Statistical comparisons were analyzed by One‐way ANOVA Tukey's multiple comparisons test. ^#^ *p* < 0.05, ^##^ *p* < 0.01, ^###^ *p* < 0.001, ^####^ *p* < 0.0001 vs MRSA group.

Encouraged by the highly potent antioxidant properties and anti‐inflammatory potential in vitro, the role of SCVN in alleviating ALI and the therapeutic effects in vivo were further evaluated. We constructed a mouse model of MRSA‐induced pneumonia and treated it with PBS, SODN, CATN, SCN, and SCVN via tracheal administration. After 7 days of administration, the lung tissues of each group of mice were pathologically analyzed by H&E staining, Masson staining, immunofluorescence staining, and inflammatory cytokine ELISA kit (**Figure** **5**a). Before studying the effect of the SCVN treatment in vivo, we firstly investigated the in vivo performance of the tracheal administration approach. We studied the biological distribution of SCVN in vivo and in different organs of mice by intratracheal injection (i.t). After rhodamine B labeled SCVN was administered by trachea at 0 h, the aggregation of SCVN in the lungs of mice was photographed by in vivo imaging system (IVIS) at 1, 6, 12, and 24 h respectively. It was found that the SCVN content gradually decreased with the extension of time (Figure 5b‐c) and was completely metabolized after 24 h, so our dosing method was determined to be daily administration. A comparison of fluorescence intensity in different organs showed that SCVN mainly accumulated in the lung, kidney, and liver (Figure S18, Supporting Information). We further evaluated the effect of SCVN on the relief of acute pneumonia tissue hypoxia. We used an oxygen probe whose fluorescence can be quenched by oxygen to evaluate the changes in oxygen in lung tissue before and after administration. As shown in Figure S19 (Supporting Information), the enzyme nanocapsule groups all showed weaker fluorescence intensity, among which SCVN showed the best fluorescence quenching effect, indicating that it can consume ROS in lung tissue through enzyme cascade reactions to generate a large amount of oxygen in situ. We further used a small animal in vivo ultrasound photoacoustic multimodal imaging system (Vevo LAZR‐X) to measure the changes in blood oxygen concentration in lung tissue before and after administration. The results also showed that SCVN can effectively increase the oxygen concentration in the lungs (Figure S20, Supporting Information) and reduce the expression of HIF‐1α (Figure S21, Supporting Information). After treatment, we measured the lung index to check for pulmonary edema. Then ELISA kits were applied to evaluate the concentration of TNF‐α, IL‐6, and IL‐1β and IL‐10 in the lung tissue. As can be seen from Figure 5d–g, compared with the PBS group, the representative pro‐inflammation cytokines TNF‐α, IL‐6, and IL‐1β in the SCVN group decreased, while the anti‐inflammatory cytokine IL‐10 increased, indicating that SCVN has in vivo anti‐inflammation and has clinical application potential for the treatment of ALI. At the same time, we assessed the inflammatory status at the histological level. H&E staining of lung tissue showed reduced congestion and decreased inflammatory cell infiltration in the treated mice (Figure 5h). Figure S22 (Supporting Information) results demonstrated that the MRSA‐induced untreated group had a higher lung index than the enzyme nanocapsules‐treated group, indicating better recovery of acute pneumonia after administration, among which SCVN showed the best therapeutic effect. Masson staining results showed that SCVN reduced MRSA‐induced pulmonary fibrosis after treatment compared with PBS administration (Figure 5h). In addition, since the production of ROS can induce the severity of lung injury, lung tissues were stained with DHE for ROS detection. Figure 5i and Figure S23a (Supporting Information) showed SCVN could significantly clear the ROS in lung tissue. In addition, the expression of M1 and M2 was evaluated at the tissue level. After SCVN treatment, the red fluorescence intensity of the M2 marker (Arg‐1) increased (Figure 5j; Figure S23b, Supporting Information), while the green fluorescence intensity of the M1 marker (iNOS) decreased (Figure 5k; Figure S23c, Supporting Information). SCVN can balance the number of M1/M2 macrophages in pneumonia tissues by inducing the transformation of pro‐inflammatory M1 macrophages to anti‐inflammatory M2 phenotype, thereby better exerting anti‐inflammatory effects and tissue repair capabilities. In addition, we also evaluated the ability of SCVN to clear lung bacteria. We homogenized the lung tissues of different treatment groups and spread them on LB agar plates. The results showed that the SCVN group had a better antibacterial effect, as manifested by a smaller number of colonies (Figure S24, Supporting Information).

**Figure 6:**
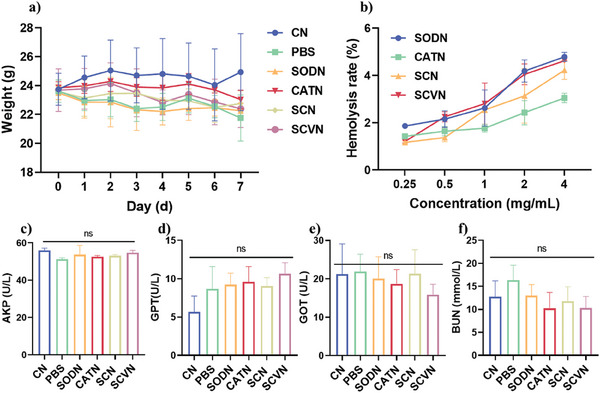
In vivo biocompatibility evaluation of SCVN. a) The mice's average body mass varies over time for various groups. b) The hemolysis rate after different nanocapsule treatments. c–f) Serum biochemical analysis in different groups. The statistical significance was acquired by one‐way ANOVA (Error bars represent mean ± s.d. (*n* = 5, ***p < 0.001, **p < 0.01, *p < 0.05, ns refers to no significance).

In addition, we further evaluated the biosafety of SCVN by testing the body weight changes, hemolysis rate, and liver and kidney functions of mice. From **Figure** 6a‐b, SCVN did not demonstrate a significant influence on the weight change of the mice and a slightly lower hemolysis rate. And as monitoring in H&E staining imaging (Figure S25, Supporting Information), the SCVN had almost no toxicity on the heart, liver, spleen, and kidney of mice, and all hematological parameters including AKP, GOT, GPT, and BUN were within the normal range after SCVN treatment in vivo (Figure 6c–f). The above results fully demonstrate that SCVN not only has good safety but also can effectively remove ROS and relieve inflammation, showing good clinical transformation prospects for the treatment of ALI.

## 3 Conclusion

Acute lung injury is usually caused by inflammatory cytokine storms and elevated ROS levels, and further upregulates the expression of HIF‐1α in immune cells, thereby releasing more pro‐inflammatory cytokines and exacerbating lung inflammatory response and oxidative stress. In summary, we successfully developed and fabricated a ROS‐driven nanoventilator based on enzyme nanocapsule with antibacterial, anti‐inflammatory, and in situ oxygen generation capabilities for the treatment of ALI caused by MRSA. This enzyme nanocapsule has high enzyme cascade activities of SOD and CAT that SOD enzyme could catalyze the conversion of SOD into H_2_O_2_ and O_2_ while CAT converts the generated H_2_O_2_ into O_2_. It is worth noting that it not only could effectively remove excess ROS but also reduce the production of inflammatory cytokines (such as TNF‐α, IL‐1β, and IL‐6) and balances the number of pro‐inflammatory M1 and anti‐inflammatory M2 macrophages through the downregulation of HIF‐1α. In addition, the introduction of vancomycin can effectively inhibit the growth of MRSA, thereby preventing bacterial proliferation from aggravating lung damage and alleviating pneumonia caused by bacteria. This nanoventilator has the characteristics of simple synthesis, safety, efficiency, and good biocompatibility, which provides a universal treatment strategy for clinical pneumonia treatment and gives it have good prospect for clinical transformation.

## 4 Experimental Section

### Materials and Instrumentation

Superoxide dismutase (SOD), Catalase (CAT), acrylamide (Acryl), N,N’‐methylene bisacrylamide, Tetramethylethylenediamine (TEMED), rhodamine B, ammonium persulfate (APS), hydrogen peroxide (H_2_O_2_), fetal bovine serum (FBS), N‐acryloxysuccinimide (NAS) were bought from Sigma. The SOD assay kit was purchased from Shanghai Zhuocai Biotechnology Co., LTD. 2,2‐Azino‐bis (3‐ethylbenzothiazoline‐6‐sulfonic acid) diammonium salt (ABTS) and Vancomycin were purchased from MACKLIN. 2‐Phenyl‐4,4,5,5‐tetramethylimidazoline‐1‐oxyl 3‐Oxide (PTIO) and tris (4,7‐diphenyl‐1,10‐phenanthroline) ruthenium (II) dichloride complex ([Ru(dpp)_3_]Cl_2_) were purchased from Bidepharm (Shanghai). iNOS antibody (CL647‐18985) and Arg‐1 antibody (CL594‐66129) were purchased from proteintech. Mouse IL‐1β (#88‐7013A‐88), IL‐6 (#88‐7064‐88), TNF‐α (#88‐7324‐88), and IL‐10 (#88‐7105‐88) uncoated ELISA kits were obtained from Invitrogen (USA, Canada). Alanine aminotransferase Assay Kit (C009‐2‐1), Urea Assay Kit (C013‐2‐1), Alkaline phosphatase assay kit (A059‐2‐2) were Aspartate aminotransferase Assay Kit (C010‐2‐1) obtained from Nanjing Jiancheng Bioengineering Institute (Nanjing, China). Phosphate‐buffered solution (PBS), Dulbecco's Modified Eagle's Medium (DMEM), and penicillin–streptomycin (PS) were purchased by Gibco. Hematoxylin and Eosin (H&E) Staining Kit, Masson Staining Kit, Trypsin (0.25%) with phenol red, DAPI‐containing sealant, 3‐(4,5‐dimethyl‐2‐thiazolyl)‐2,5‐diphenyl‐2‐H‐tetrazolium bromide (MTT) were purchased from Yeasen. DCFH‐DA was obtained from Beyotime Biotechnology. Yeast extract powder and tryptone were obtained from OXOID. Agar powder was purchased from Solarbio.

### Preparation and Characterization of SCVN

The synthesized SCVN was based on our previously reported method.^[^ ^11^ ^]^ Briefly, Acryl was added to the solution of acryloylated SOD and CAT. The ultimate protein levels were attenuated to 1 mg mL^−1^ utilizing 1x PBS. N, N’‐methylene bisacrylamide, and Acryl as crosslinkers were dissolved by DMSO, and then Vancomycin was dropped into the mixing solution. Finally, the initiator APS and the catalyst TEMED were dripped into the aforementioned mixture to trigger polymerization under the nitrogen atmosphere. The final nanocapsule products were obtained after 2 h of stirring reaction at RT condition and then received dialysis and lyophilization to remove excess monomers, initiators, and the relevant solvent.

### Evaluation of Superoxide Anion (•O_2_^−^) Removal Rate (SOD Activity) of SOD‐CAT Nanocapsule (SCN)

The enzyme activity of SCN was determined by an enzyme‐specific method. Taking SOD as an example, superoxide anion (•O_2_^−^) was prepared by xanthine and xanthine oxidase (XOD) as the substrate of SOD, and reacts with WST‐8 to generate formaldehyde dye with high‐water solubility, absorbing at 450 nm.^[^ ^18^ ^]^ SOD can eliminate •O_2_^−^, thus precluding the formation of formazan. When the yellow color of the reaction solution gets deeper, it indicated that the compound had a lower SOD activity. Briefly, 10 µL 0.1 mg mL^−1^ SOD solution, 180 µL WST operating fluid, and 10 µL XOD enzyme operating fluid (diluted with 1:10 diluent) were added according to a certain order. After being thoroughly mixed and standing at room temperature for 30 min, the absorption value of each tube was measured at 450 nm. In the control group, the sample solution was replaced with distilled water, and the SOD activity was evaluated with the percentage of inhibitory formazan formation.

### Evaluation of Dissolved Oxygen Generation Rate (CAT Activity) of SCN

Several kinds of assessments, including 30% H_2_O_2_, 30% H_2_O_2_ + SODN, 30% H_2_O_2_ + CATN, and 30% H_2_O_2_ + SCN were set up for the purpose of confirming the reacting capacity of CAT and the entire system. The JPB‐607A portable dissolved oxygen tester was applied to measure the oxygen levels of each group at several points in time while they were maintained in an incubator at 37 °C.

### •OH Scavenging Ability of SCN

Free radicals exert a detrimental influence on biological systems, hence the capacity of eliminating free radicals was essential for compounds to shield macromolecules from oxidative damage. Therefore, The •OH scavenging ability of SCN was determined through a salicylic acid method.^[^ ^19^ ^]^ Briefly, 0.1 mg mL^−1^ of different types of nanocapsules, FeSO_4_, H_2_O_2_, and salicylic acid solutions were mixed in a certain order and incubated at 37 °C for 30 min. The full wavelength scan curve of this solution was determined and its absorbance at 510 nm was observed.

### ABTS^•+^‐Scavenging Ability of SCN

ABTS^•+^ may be produced by oxidizing ABTS. The blue‐green solution possesses a distinctive absorption peak at 734 nm.^[^ ^20^ ^]^ Briefly, samples were mixed with 20 µL ABTS working solution and 1 mL PBS, allowing them to undergo reaction in the dark overnight. Then, the whole wavelength scanning curves were estimated, and the absorbance statistics at 734 nm were recorded.

### PTIO•‐Scavenging Ability of SCN

The final concentration of PTIO• free radical was 143 µM in 3 mL ultra‐pure water, and after diluting the mother solution 10 times, take 200 mL diluted solution and add to different amounts of nanocapsule. Following incubation at 25 °C without light for 2 h, the absorption spectrum of the mixture was recorded.^[^ ^21^ ^]^

### Cell Culture

Mouse mononuclear macrophage leukemia cells (RAW 264.7) were kindly cultured with DMEM adding 10%–20% sigma FBS under the condition of 37 °C and 5% CO_2_ in the cell incubator.

### Cytotoxicity Assay

The RAW 264.7 cells (1.0 × 10^4^ cells/well) were placed in 96‐well plates and allowed to develop overnight. Afterward, the cells were administered with several concentrations of the SCN compound for a duration of 24 h. The medium with MTT was added to the plate following a 4‐h incubation period. Subsequently, an absorbance microplate reader with UV absorption at 490 nm was implemented to quantify the viability of the RAW 264.7 cells administrated with different prepared composites.

### Live/Dead Staining Assay

RAW 264.7 cells were stained both alive and dead employing a Calcein‐AM/PI double stain kit. The RAW 264.7 cells (5 × 10^4^ cells/well) were plated onto 24‐well plates and allowed to develop overnight. Afterward, the cells were exposed to 100 µM H_2_O_2_ for 12 h. Then, the cells were administered with 50 µg mL^−1^ SODN, CATN, and SCN for 24 h. The cells were thoroughly rinsed with 1 × assay buffer before being cultured for 15 min using the live/dead working solution. By applying a fluorescent microscope, it can easily distinguish the live and dead cells with yellow‐green and red fluorescence, respectively.

### Intracellular ROS Elimination and O_2_ Generation

The capacity of generating O_2_ and scavenging intracellular ROS was evaluated utilizing RAW 264.7 cells treated with SCN. 12‐well plates were incubated with RAW 264.7 cells overnight, then cultured with FBS‐free DMEM and SCN mixture for 24 h to assess the ROS inhibition. Afterward, the ROS probe (DCFH‐DA) was added into the 2 mL centrifuge tube containing cells collection for to 30 min. After observing the fluorescence with an inverted fluorescence microscope (Dmi8, Leica, Germany), ImageJ software was used to quantify the mean fluorescence. For intracellular O_2_ investigation, after being seeded on a 12‐well plate for the night, the cells were treated for 6 h with 100 µM H_2_O_2_ and H_2_O_2_ + SCN composite (50 µg mL^−1^ in serum‐free DMEM). Subsequently, the cells were subjected to a 6‐h treatment with 10 µg mL^−1^ of [Ru(dpp)_3_]Cl_2_, and to remove any remaining [Ru(dpp)_3_]Cl_2_ after PBS rinsing 3 times. ImageJ software was employed to measure the fluorescence after it was photographed using an inverted fluorescent microscope (Dmi8, Leica, Germany).

### Enzyme‐Linked Immunosorbent (ELISA) Assay of Cells

The RAW 264.7 cells at a density of 5 × 10^5^ cells per well were seeded in 6‐well plates and adhered 24 h. The cells were treated for 12 h with H_2_O_2_ (100 μM) and H_2_O_2_ + SODN, CATN, or SCN (100 µg mL^−1^). The supernatant was gathered and determined by ELISA.

### Immunofluorescence of Phenotypic Analysis of Macrophages

RAW 264.7 cells were used to evaluate the functions of SCN complexes on phenotypic changes in macrophages. The cells (1 × 10^4^ cells/well) were planted into 48‐well plates with slides overnight and the cells were completely attached to the wall and incubated with the FBS‐DMEM‐free SCN complex (50 µg mL^−1^, FBS‐DMEM‐free). After 12 h, the cells were incubated with M1 marker FITC‐inducible nitric oxide synthase (iNOS) antibody (1:100) (Ex = 493 nm, Em = 522 nm) and antibody to M2 marker PE‐Arginase‐1 (Arg‐1) (1:100) (Ex = 588 nm, Em = 604 nm) overnight at 4 °C. After that, it was rinsed 3 times with PBS to eliminate the staining solution and then immobilized with 4% paraformaldehyde. Finally, DAPI was unutilized to dye and seal the flakes. High‐sensitivity confocal laser microscope Zeiss LSM5 was employed to detect the images.

### Real‐Time Fluorescence Quantitative PCR (qPCR) Analysis

Following a 12h incubation period with H_2_O_2_ (100 μM) and different enzyme nanocapsules, total RNA was collected with Tricom reagent. The NanodropND‐1000 spectrophotometer was utilized to measure the quantity and quality of RNA. The total RNA was then reverse‐transcribed into cDNA utilizing Hifair Il 1st Strand cDNA Synthesis SuperMix for qPCR (gDNA digester plus) with gDNA eraser. The primer sequence is displayed in **Table** **1**. In the final, Hieff UNICON Universal Blue qPCR SYBR Green Master Mix was employed to carry out the qPCR. The heat cycle settings were 45 cycles of 15 s at 95 °C, 30 s at 95 °C, and 15 s at 60 °C.

**Table 1:** Primer sequences used in the experiment.

Gene	Primer sequence
GADPH	5’‐ATGTGTCCGTCGTGGATCTGA‐3’ 5’‐TGCCTGCTTCACCTTCT‐3’
iNOS	5’‐GATGTTGAACTATGTCCTATCTCC‐3’ 5’‐GAACACCACTTTCACCAAGAC‐3’
Arg‐1	5’‐CAAGACAGGGCTCCTTTCAG‐3’ 5’‐TGGCTTATGGTTACCCTCCC‐3’

### Immunofluorescence of Expression of Hypoxia‐Inducible Factor‐1α (HIF‐1α)

12‐well plates were incubated with RAW 264.7 cells overnight, then cultured with H_2_O_2_ (100 μM) and H_2_O_2_ + SODN, CATN, SCN and SCVN (100 µg mL^−1^). And then 10 µg mL^−1^ deferoxamine in each well was added to induce the expression of HIF‐1α and cultured with the anaerobic bags to create hypoxia condition. After 24 h the cells were incubated with hypoxia‐inducible factor‐1α(HIF‐1α) antibody (EPR16897) overnight at 4 °C. After that, it was rinsed 3 times with PBS to eliminate the staining solution and conjugated with FITC‐rabbit antibody for 2 h, and then immobilized with 4% paraformaldehyde. Finally, DAPI was unutilized to dye and seal the round coverslips. High‐sensitivity confocal laser microscope Zeiss LSM5 was employed to detect the images.

### Western Blotting

12‐well plates were incubated with RAW 264.7 cells overnight, then cultured with H_2_O_2_ (100 μM) and H_2_O_2_ + SODN, CATN, SCN and SCVN (100 µg mL^−1^). And then 10 µg mL^−1^ deferoxamine in each well was added to induce the expression of HIF‐1α and cultured with the anaerobic bags to create hypoxia condition. After 24 h the cell proteins were extracted by using a loading buffer and a BCA protein kit was used to determine the concentration of proteins. The denatured protein was separated by 7.5% SDS‐PAGE gels and transferred to the PVDF membrane. After blocking the PVDF membrane with 5% nonfat milk for 2 h, the PVDF membrane was incubated with Anti‐HIF‐1 alpha antibody [EPR16897] (1:10000) and the β‐actin antibody (1:10000) overnight at 4 C. After washing with TBST and incubated with secondary antibody (1:5000) for 2 h at room temperature. The protein band signals were detected with Super Signal West Femto maximum sensitivity substrate (Yeasen) under visualization in a ChemiDoc MP Imaging System (Bio‐Rad, Hercules, CA, USA).

### Antibacterial Activity of SCVN In Vitro

The antibacterial activity of the SCVN complex was detected by Methicillin‐resistant *Staphylococcus aureus* (MRSA). The suspension was diluted with sterile LB medium and the absorbance at 600 nm was OD 0.1. SCVN solutions of different concentrations (3 and 7 mg mL^−1^) were added with 50 µL diluted bacterial solution, then LB medium was used as the control, and fully shaken in an incubator at 37 °C with no less than 90% relative humidity for 12 and 36 h, respectively. Ultimately, the absorbance of the different groups was assessed at 600 nm. To more directly observe the antibacterial effect of the material, the material was mixed with bacterial solution (50 µL OD 0.05) and evenly coated on the surface of LB agar placed for 12 h, and the live bacteria were observed and photographed.

### Bacterial Activity/Toxicity Test

A NucGreen/EthD‐III double stain kit was employed to distinguish the living and death of bacteria. 1 volume of component A NucGreen and 2 volumes of component B EthD‐III were mingled in a microcentrifuge tube. After the full mixture, 8 volumes of 0.85% NaCl solution were appended to acquire 100× stain solution. 1 µL of 100× stain solution was added to 100 µL bacterial suspension and every mixing well was cultured at room temperature under dark conditions for 15 min. The 5 µL stained bacterial suspension drops were observed under a fluorescence microscope in which living bacteria had green or yellow‐green fluorescence while dead bacteria had red fluorescence.

### MRSA‐Induced Acute Lung Injury of Mice

C57BL/6 mice were obtained from Xiamen University Laboratory Animal Center and all animal experiments were approved by the Animal Management and Ethics Committee of Xiamen University (XMULAC20210116). Six groups (*n* = 5 each) were established from all of the mice: Control group (without treatment),ALI model group (given PBS), SODN group (5 mg mL^−1^, i.t.), CATN group (5 mg mL^−1^, i.t.), SCN group (5 mg mL^−1^, i.t.), SCVN group (5 mg mL^−1^, i.t.). In addition to the control group, the other groups were administrated with MRSA (OD 0.1, 50 µL, i.t.) instillation into the lungs of mice. After being treated with MRSA 72 h, the mice were administrated with different prepared nanocapsules within 7 days. Parts of lung tissues were collected for measurement of pro‐inflammation and anti‐inflammation cytokines. Other parts of lung tissues were obtained for histological analysis and immunofluorescence staining. Besides, the lung tissues were obtained the weight was recorded, and the lung index of the mice was calculated according to Table S1 and Figure S22, Supporting Information).

### Measurement of Oxygen Content in Lung Tissue

We used an oxygen probe (Platinum(II) meso‐Tetra(pentafluorophenyl)porphine) to determine the difference in lung oxygen concentration in mice with MRSA‐induced acute pneumonia treated with different enzyme‐containing nanocapsules. The method was usedin 4.15 to establish a MRSA‐induced pneumonia mouse model. After the model was established, an oxygen probe (50 µL, 1 mg mL^−1^) was added through tracheal administration. After 30 min, 50 µL of 5 mg mL^−1^ CATN, SODN, and SCVN were administered through the trachea, and normal saline was used as a control. After another 30 min, the small animal in vivo imaging system (Caliper IVIS Lumina II) was used to monitor the lung oxygen concentration in mice.

In addition, the lung blood oxygen content of MRSA‐induced pneumonia mice after drug administration using a small animal photoacoustic imaging system (Vevo LAZR‐X) was further observed. All operations were performed as above except that the oxygen probe was not added. Finally, the small animal photoacoustic imaging system was used to observe and record the changes in the lung oxygen content of mice.

### Enzyme‑Linked Immunosorbent (ELISA) Assay of Lung Tissues

Fresh lung sections were dissected and stored in PBS. After rinsing with PBS, lung tissues were homogenated and centrifuged for 10 min at 2000 × g, and the supernates were subsequently conserved at −20 °C. The concentration of TNF‐α, IL‐6, IL‐1β, and IL‐10 in lung tissue were detected using ELISA kits (Invitrogen, Thermo Fisher Biotechnology) in accordance with the manufacturer's instructions.

### Detection of ROS Levels in Mice Lung Tissue

Frozen sections of lung tissue were taken, using PBS to rinse 3 times within 15 min, and wiped dry, PBS containing DHE (Beyotime Biotechnology) was added dropwise onto the slides, and incubated for 30 min at 37 °C. The slices were stained with DAPI to locate the cell nuclei after 3 PBS washes. Then the fluorescence intensity in lung tissue was observed by fluorescence microscopy to represent the level of ROS in mice lung tissue.

### Histological Analysis

To prepare for the histological investigation, tissues from the lung, kidney, liver, spleen, and heart were removed and submerged for 24 and 12 h, respectively, in a solution containing 15% and 30% sucrose. Issue segments were submerged in an optimal cutting temperature (OCT) compound and cut into 6 µm‐diameter tissue pieces using a freeze slicer (CM1900, Leica, Germany). They were examined and analyzed under a microscope following staining with hematoxylin and eosin (H&E) and Masson's Trichrome dye kit (Solarbio).

### Immunofluorescence Staining

Lung tissue fragments were frozen into slices in preparation for fluorescence immunostaining after being dehydrated. The slice was treated with ice‐cold acetone 20 min, washed in PBS, and blocked for 1 h using a 5% Bovine Serum Albumin (BSA) solution. The lung slice was incubated with M1 marker FITC‐inducible nitric oxide synthase (iNOS) antibody (1:100) (Ex = 493 nm, Em = 522 nm) and antibody to M2 marker PE‐Arginase‐1 (Arg‐1) (1:100) (Ex = 588 nm, Em = 604 nm) at 4 °C overnight to verify the anti‐inflammatory effects in vivo. The staining solution was then removed from the sections by continually rinsing them with PBS. Ultimately, the slices underwent DAPI staining and confocal microscopy imaging.

### Statistical Analysis

The graphs and experimental data were all computed with GraphPad Prism 8.0. The results were displayed as mean ± standard deviation (SD), and the t‐test was used to examine the distinctions between various groups. *p < 0.05, **p < 0.01, and ***p < 0.001 vs the designated group were deemed as statistically noticeable variations when the P‐value was less than 0.05.

## Conflict of Interest

The authors declare no conflict of interest.

## Supporting information



Supporting Information

## Data Availability

The data that support the findings of this study are available from the corresponding author upon reasonable request.
