# Supplementary material for: ROS‐Driven Nanoventilator for MRSA‐Induced Acute Lung Injury Treatment via In Situ Oxygen Supply, Anti‐Inflammation and Immunomodulation
Source: Adv Sci (Weinh). 2025 Mar 19;12(18):2406060. doi: 10.1002/advs.202406060 (PMC12079454; doi:10.1002/advs.202406060)
Supplement: Supplementary file 1 — Supporting Information [file ADVS-12-2406060-s001.docx]

Supporting Information

for

**ROS-Driven Nanoventilator for MRSA-Induced Acute Lung Injury Treatment via In Situ Oxygen Supply, Anti-Inflammation and Immunomodulation**

Zheng Luo^1,2,§^, Qi Wang^1,^^§^, Xiaotong Fan^3,§^, Xue Qi Koh^2^, Xian Jun Loh^2^, Caisheng Wu^1^, Zibiao Li^2,3,4,^* and Yun-Long Wu^1,^*

1 State Key Laboratory of Cellular Stress Biology, Fujian Provincial Key Laboratory of Innovative Drug Target Research, School of Pharmaceutical Sciences, Xiamen University, Xiamen 361102, China

2 Institute of Materials Research and Engineering (IMRE), Agency for Science, Technology and Research (A*STAR), 2 Fusionopolis Way, Innovis #08-03, Singapore 138634, Republic of Singapore

3 Institute of Sustainability for Chemicals, Energy and Environment (ISCE2), Agency for Science, Technology and Research (A*STAR), 1 Pesek Road, Jurong Island, Singapore 627833, Republic of Singapore

4 Department of Materials Science and Engineering, National University of Singapore, Singapore, 117576, Republic of Singapore

*Correspondence to: [lizb@imre.a-star.edu.sg](mailto:lizb@imre.a-star.edu.sg) (Z. Li); and [wuyl@xmu.edu.cn](mailto:wuyl@xmu.edu.cn) (Y-L. Wu)

§These authors contribute equally to this work

**
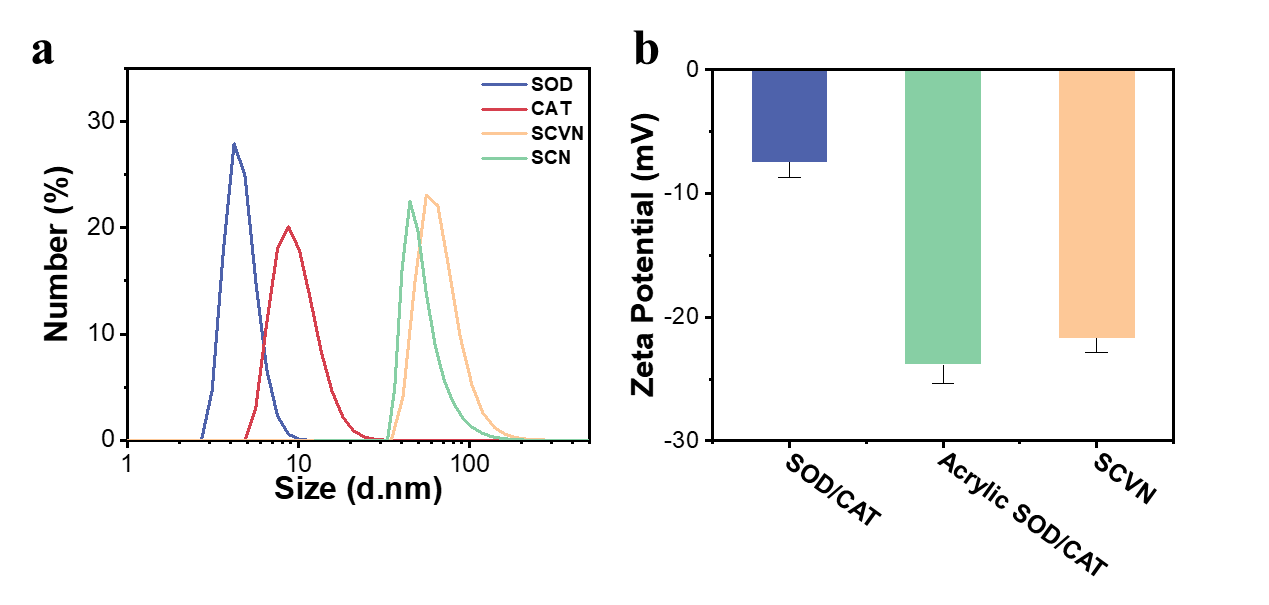
**

**Figure S1** a) DLS analysis of SOD, CAT, SCN and SCVN. b) Zeta potential of the mixture of SOD and CAT, the mixture of acrylic SOD and CAT, and the enzyme nanocapsules SCVN.

**
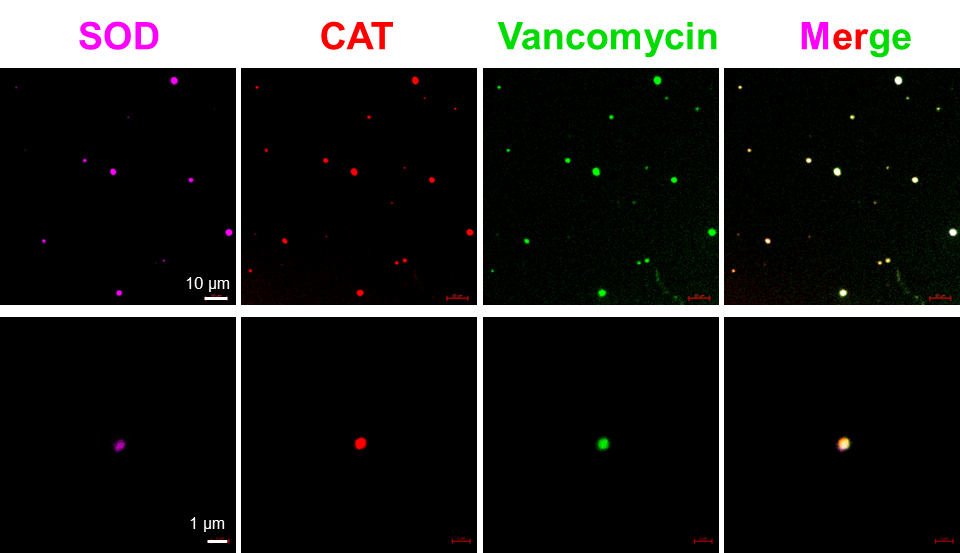
**

**Figure S2** Confocal images of nanocapsules formed by *in-situ* polymerization of SOD, CAT and vancomycin modified with Cy3.5, Rhodamine B and FITC, respectively.

**
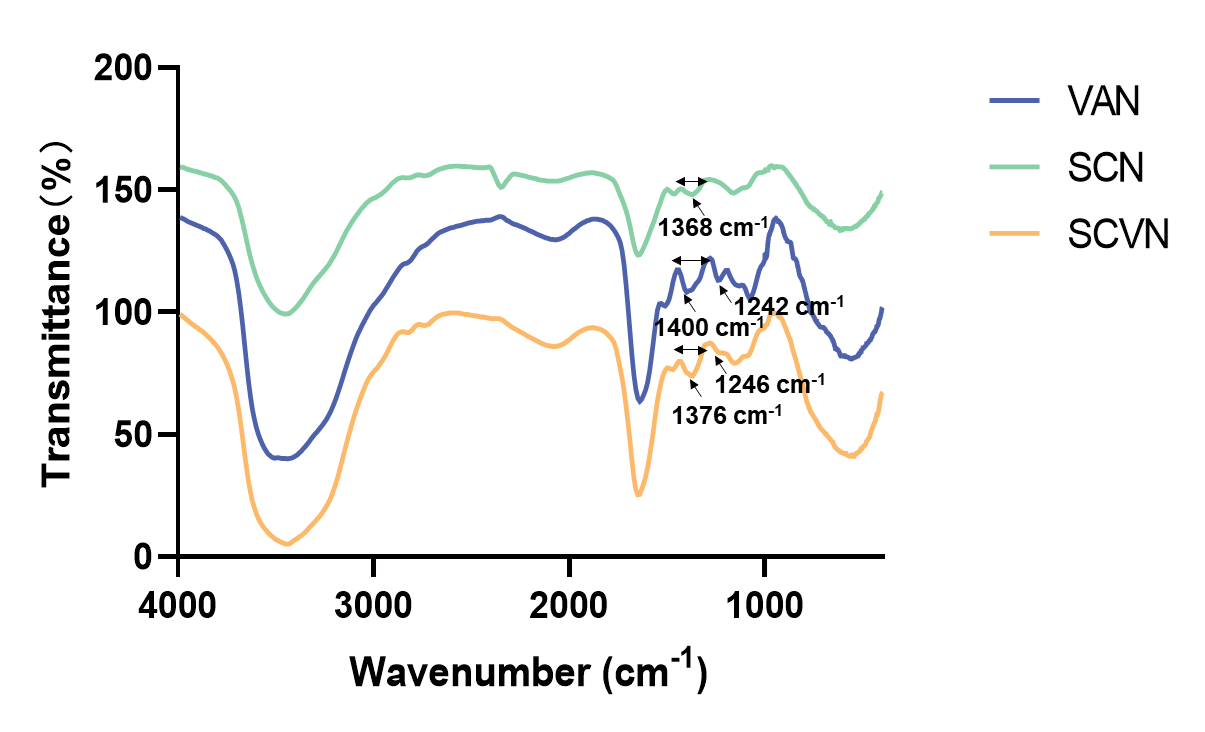
**

**Figure S3** FTIR spectrum of vancomycin (VAN), SCN, and SCVN.

**
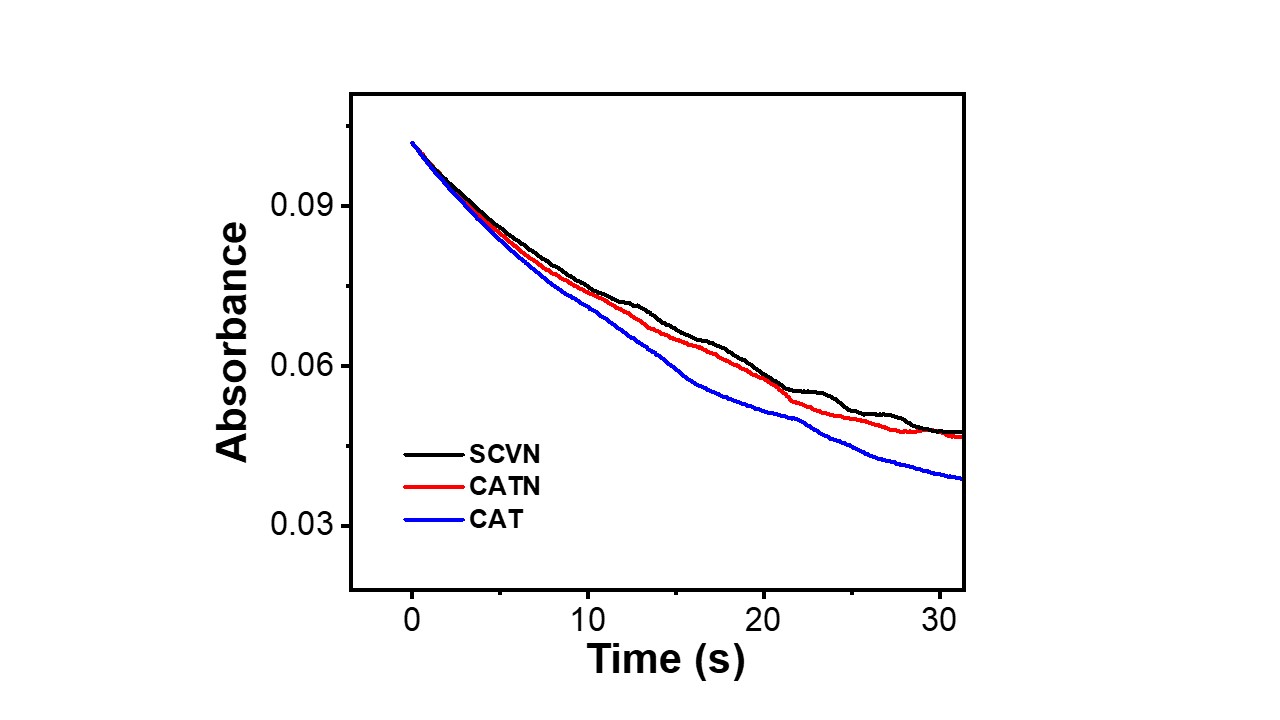
**

**Figure S4** Comparison of CAT activity for native CAT, CAT nanocapsules (CATN) and SOD-CAT/Vancomycin nanocapsules (SCVN). The test result indicated the catalytic activity of SCVN kept 84.5% of native CAT activity, CATN kept 91.6% of the native CAT activity.


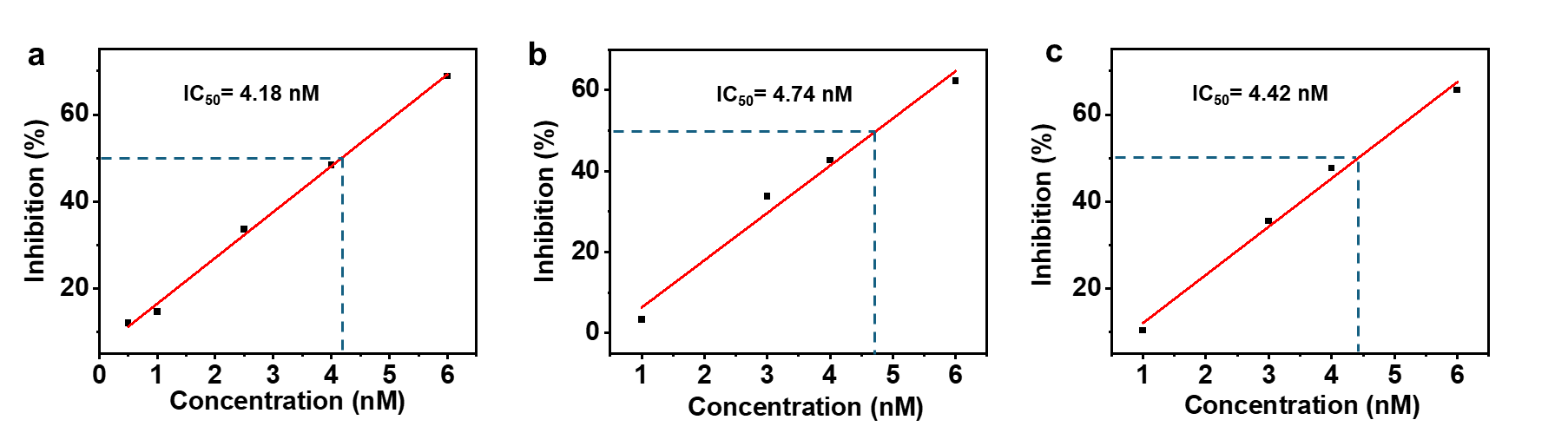


**Figure S5** Percentage of inhibition of NBT oxidation by superoxide anion radical *versus* different concentrations of a) native SOD, b) SOD nanocapsule (SODN), and c) SOD-CAT/Vancomycin nanocapsule (SCVN). The examination results indicated the catalytic activity of SODN kept 88.2% of native SOD activity, SCVN kept 94.5% of the native SOD activity.


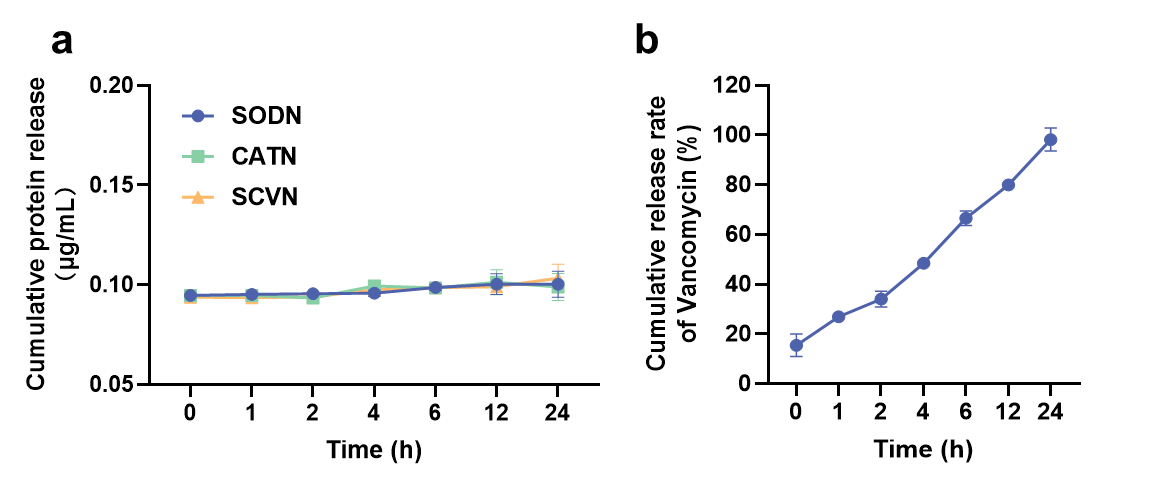


**Figure S6** At pH = 6.8, a) the *in vitro* release behavior of enzymes from SODN, CATN and SCVN, b) the *in vitro* release behavior of vancomycin from SCVN.





**Figure S7** Loss of activity of CAT in different enzyme forms after 30 minutes in a water bath under 60^o^C.


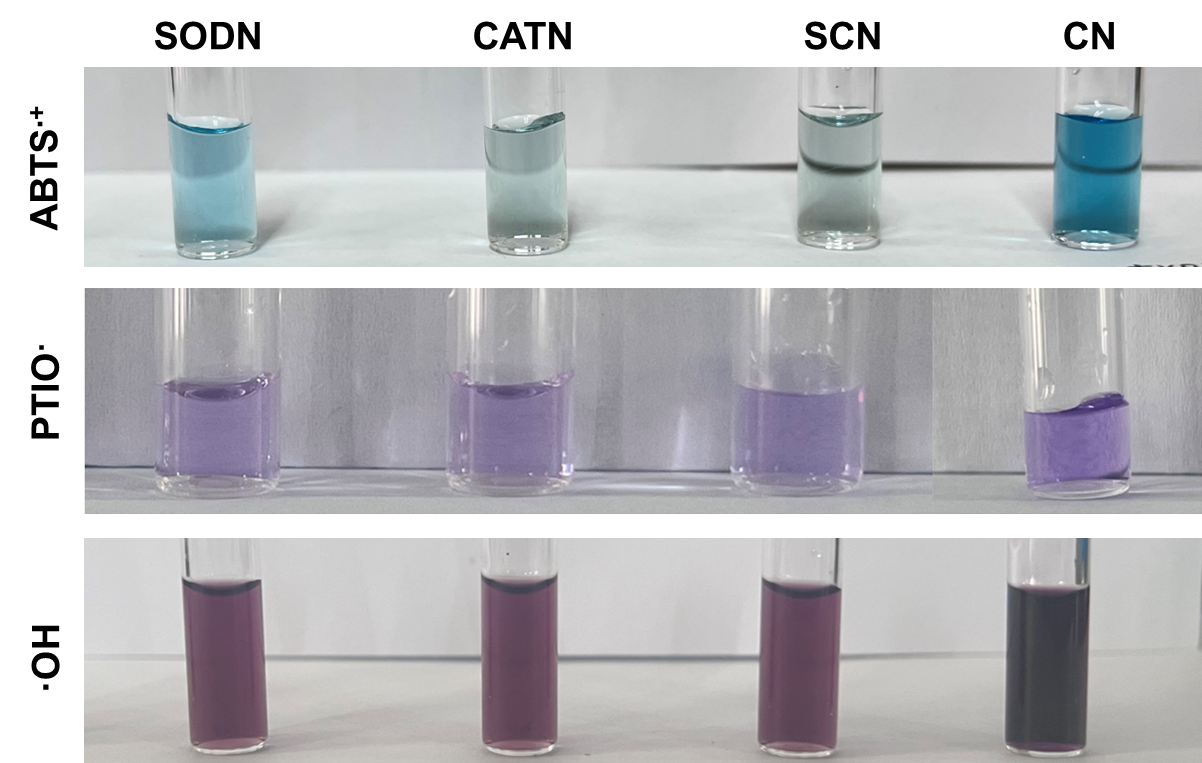


**Figure S8** Color changes of different materials after interaction with ABTS^•+^, PTIO• and •OH.

**Figure S9** Quantitative analysis of ROS content in Figure 2a (n=3).


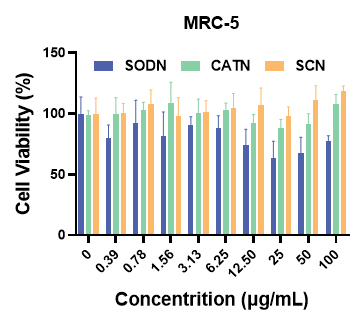


**Figure S10** Cytotoxicity of different enzyme nanocapsules on human embryonic lung fibroblasts cells (MRC-5).


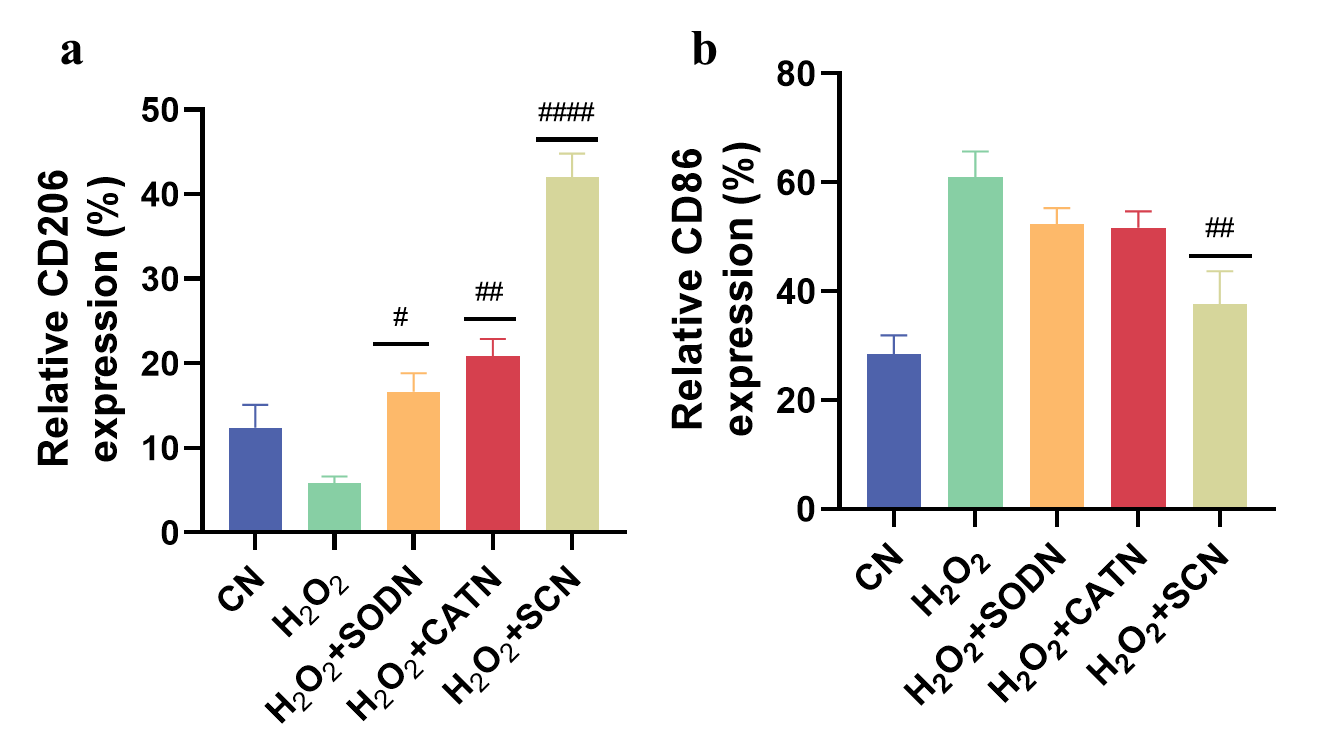


**Figure S11** Quantitative analysis of the expression of (a) CD206 and (b) CD86 in Figures 3a and 3b, respectively.


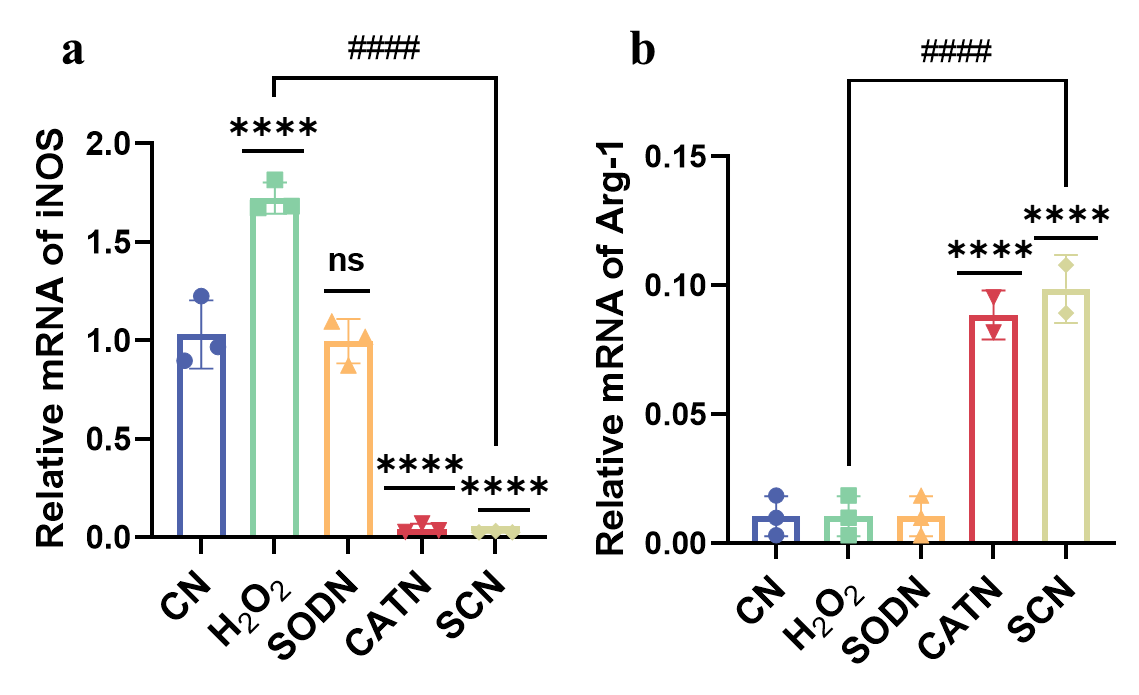


**Figure S12** mRNA expression of a) iNOS and b) Arg-1 markers in RAW 264.7 cells by qPCR (n=3).


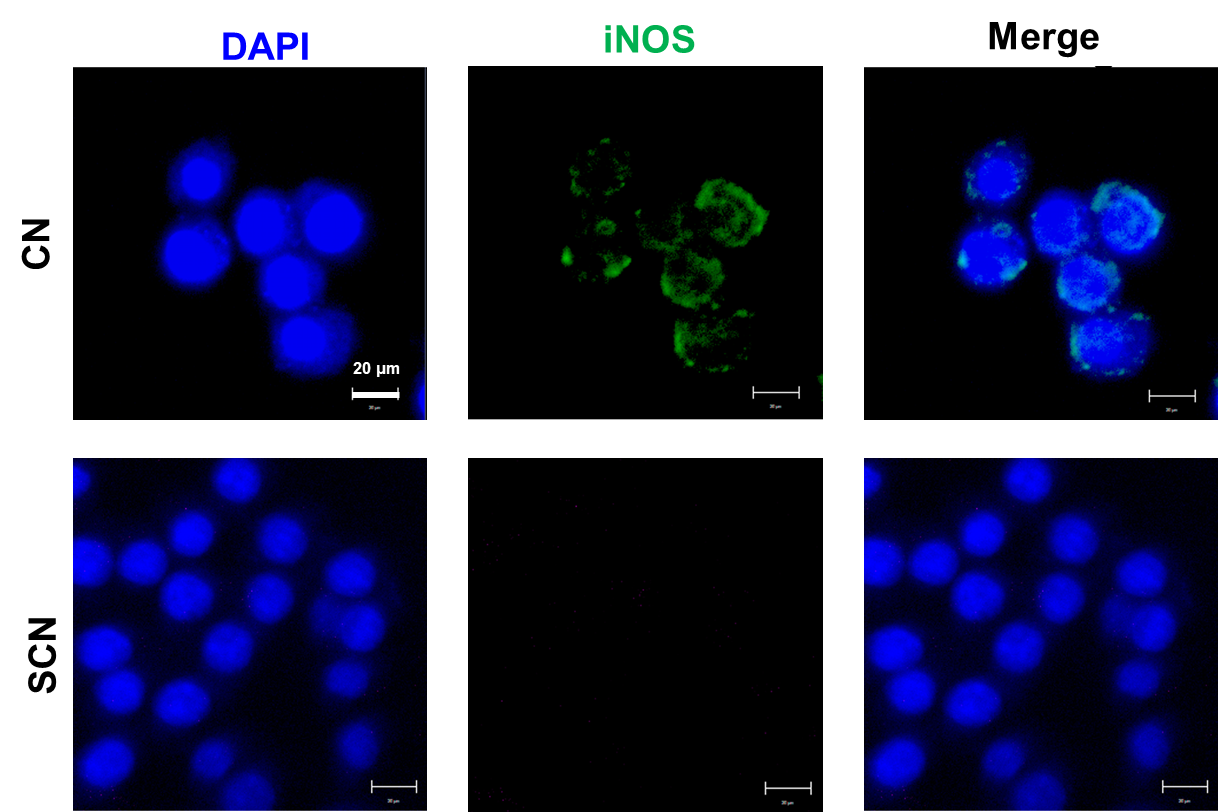


**Figure S13** Representative images of M1-associated marker (iNOS) expression of RAW 264.7 cells cultured for 24 h under different conditions. Scale bar: 20 µm.


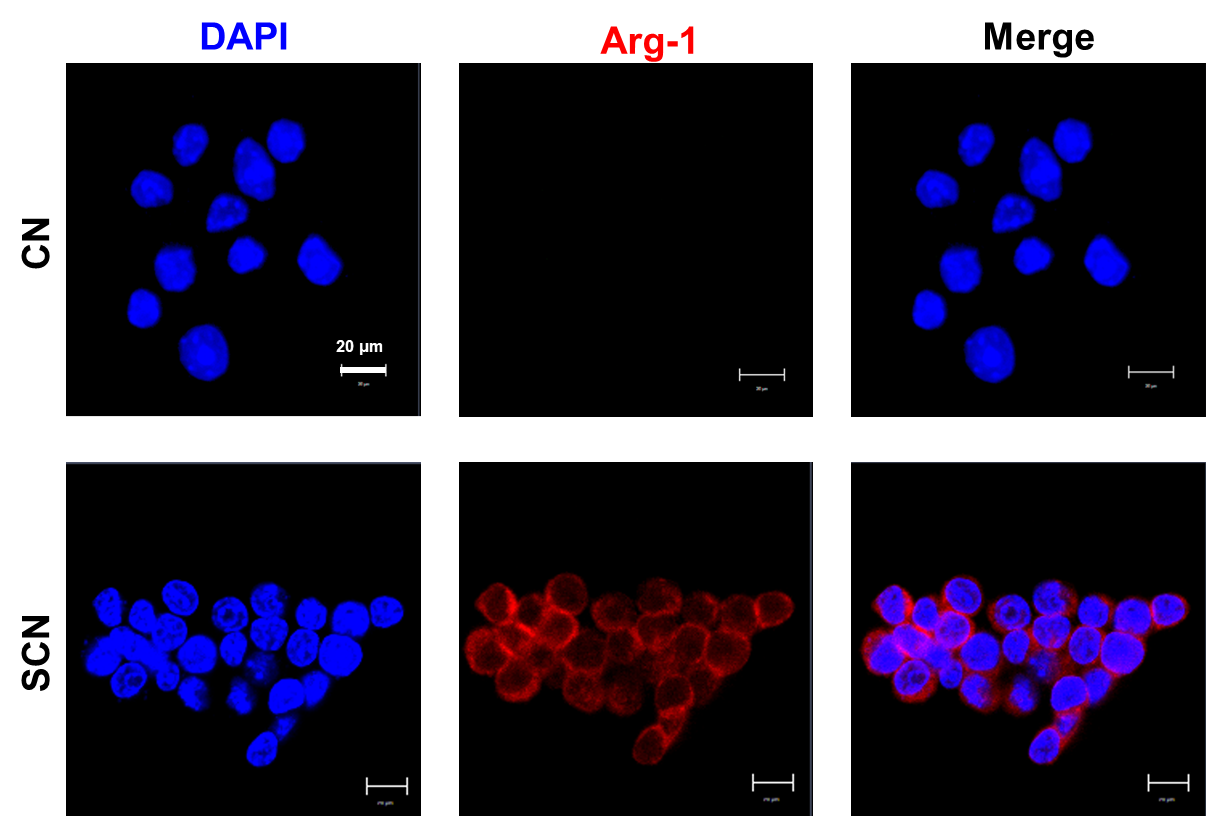


**Figure S14** Representative images of M2-associated marker (Arg-1) expression of RAW 264.7 cells cultured for 24 h under different conditions. Scale bar: 20 µm.


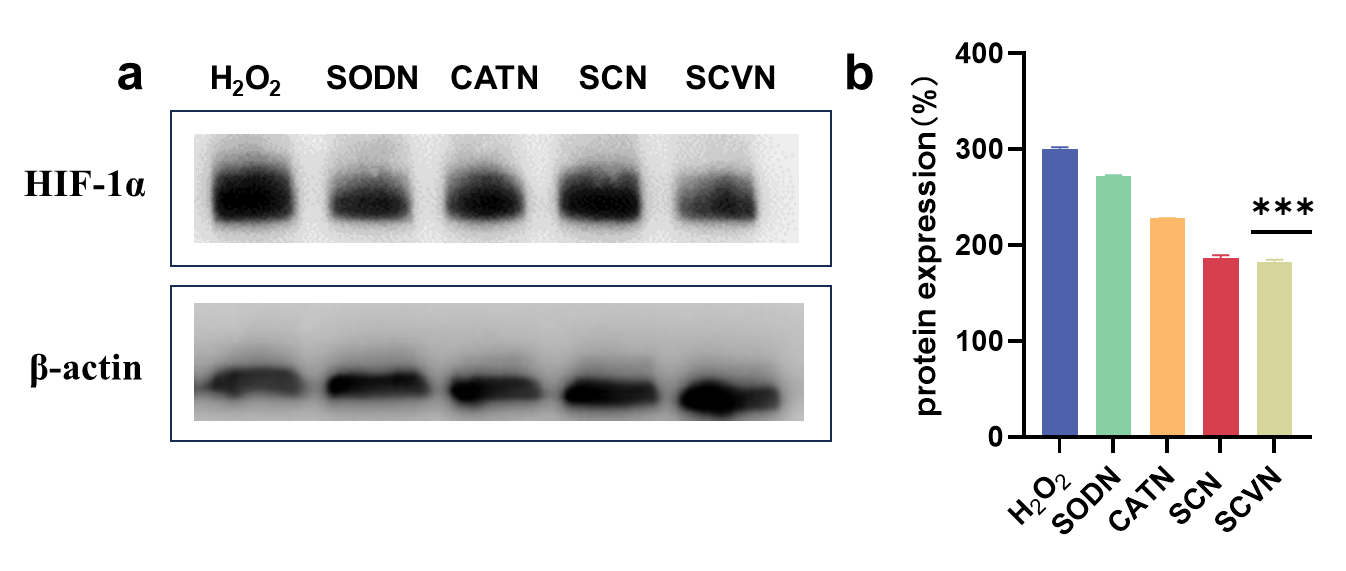


**Figure S15** Hypoxia-induced expression of HIF-1α detected by Western blotting assay.

**Figure S16** Growth curve of MRSA within 36 hours after adding different SCVN concentrations.


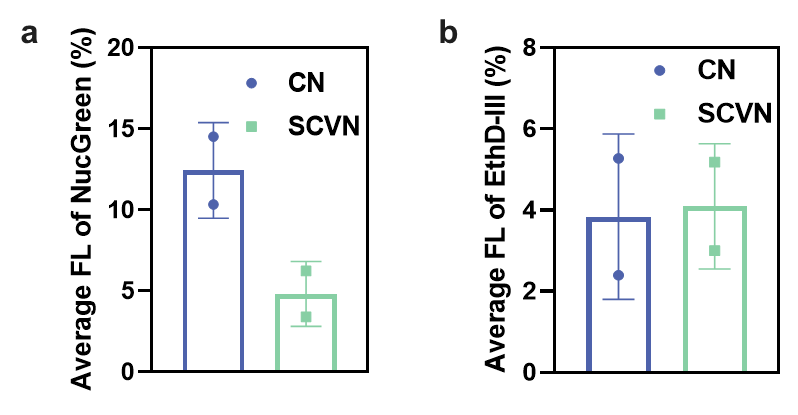


**Figure S17** a) and b) The quantitative studies of green and red fluorescence of bacteria live/dead staining using ImageJ software (n=2).


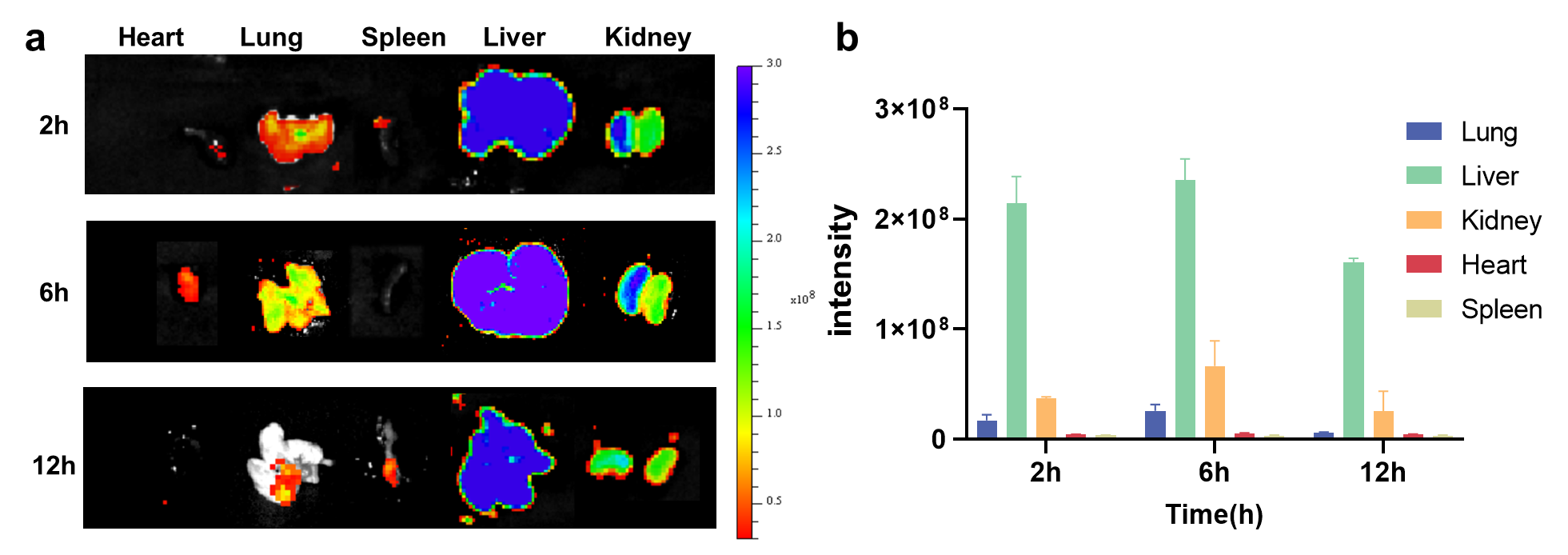


**Figure S18** The distribution of SCVN in different organs after different time points (2 h, 6 h,12 h).


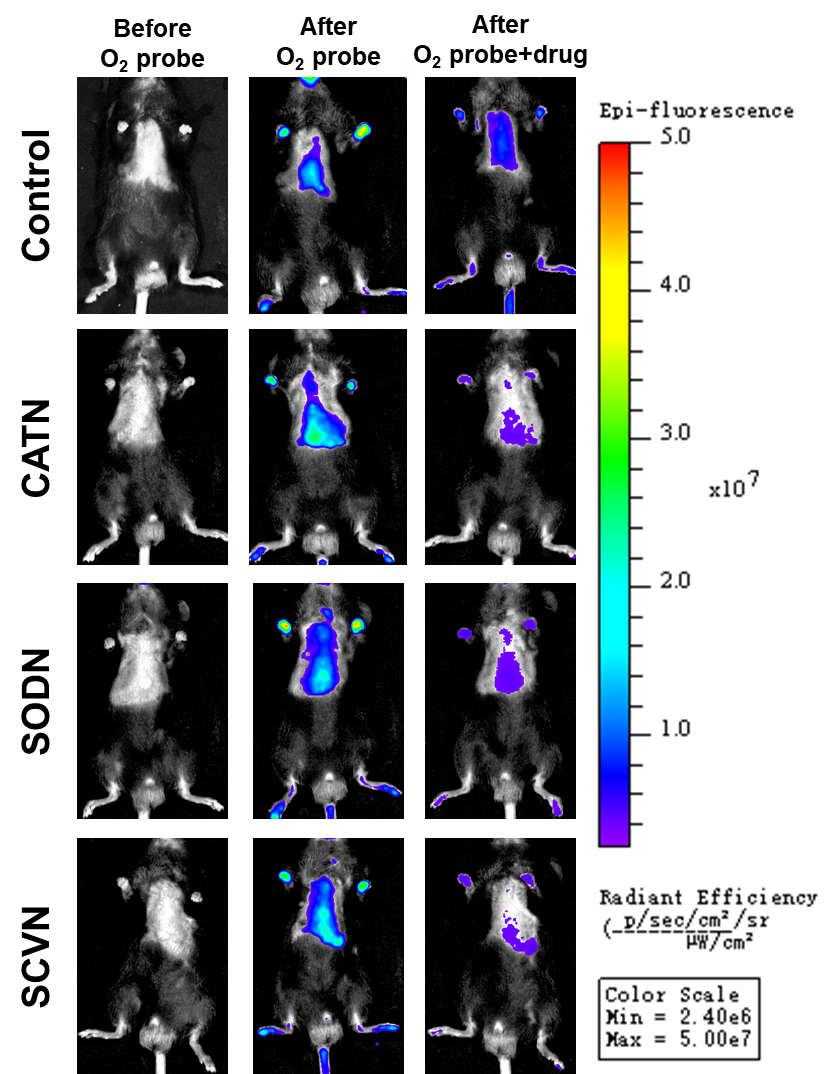


**Figure S19** The O_2_ probe (Platinum(II) meso-Tetra(pentafluorophenyl)porphine) was used to evaluate the effects of different nanocapsules on alleviating lung hypoxia in MRSA-induced acute pneumonia tissues.


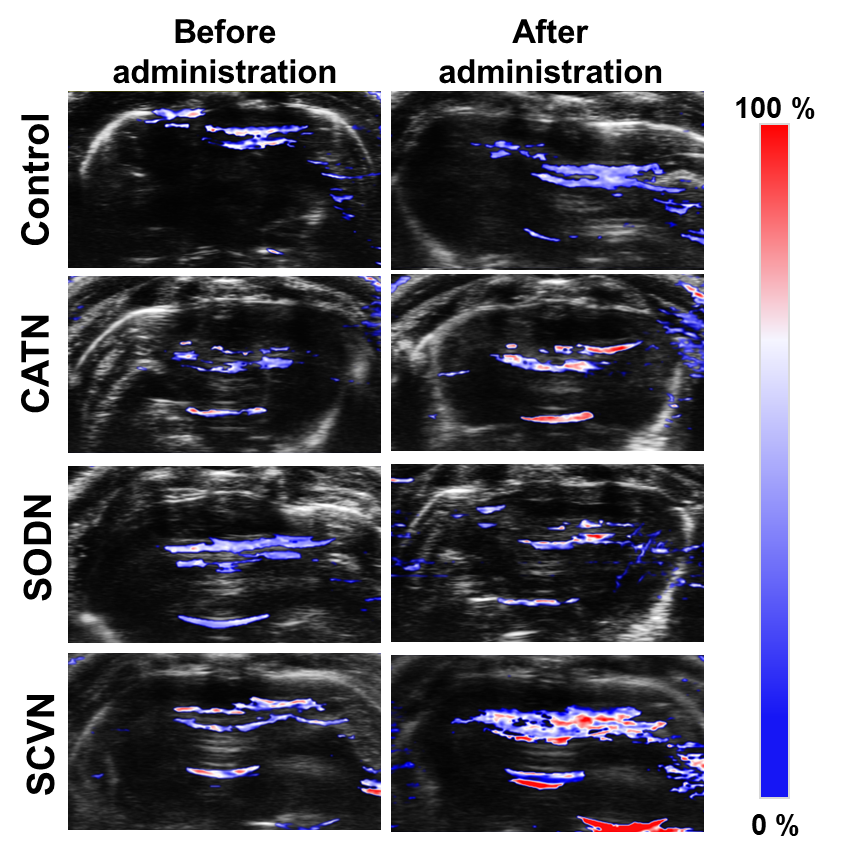


**Figure S20** Photoacoustic blood oxygen imaging of MRSA-induced acute pneumonia tissues treated with different nanocapsules.


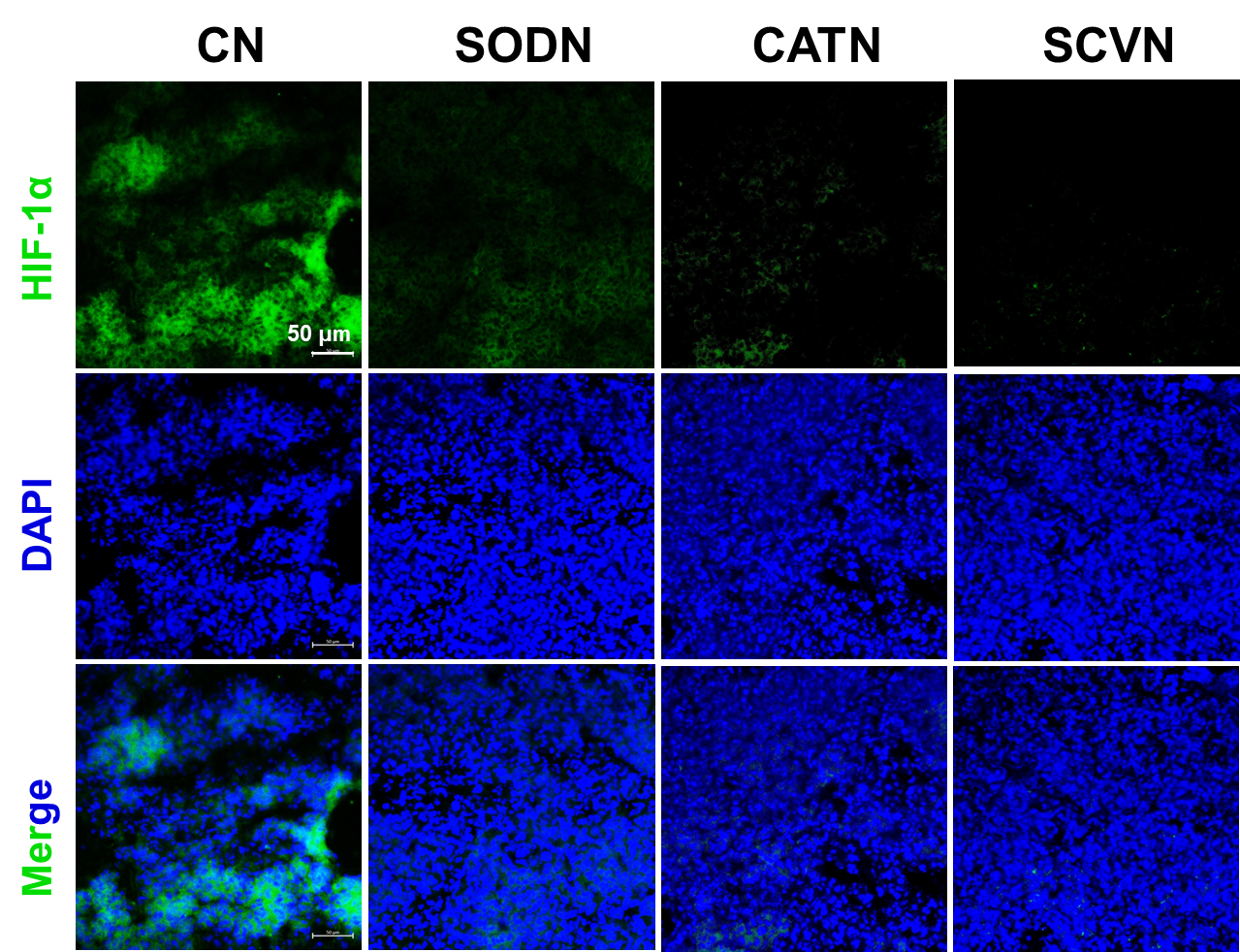


**Figure S21** Immunofluorescence staining of HIF-1α in tissues after treatment with different nanocapsules.

Table S1. Lung injury scoring system

|  | Score per field | | |
| --- | --- | --- | --- |
| **Parameter** | **0** | **1** | **2** |
| A. Neutrophils in the alveolar space | none | 1-5 | >5 |
| B. Neutrophils in the interstitial space | none | 1-5 | >5 |
| C. Hyaline membranes | none | 1 | >1 |
| D. Proteinaceous debris filling the airspaces | none | 1 | >1 |
| E. Alveolar septal thickenings | <2× | 2×-4× | >4× |





**Figure S22** Total lung injury score obtained from Table S1 (Score=[(20*A) +(14*B)+(7*C)+(7*D)+(2*E)]/(number of fields*100)) primarily based on the H&E images (n = 3).


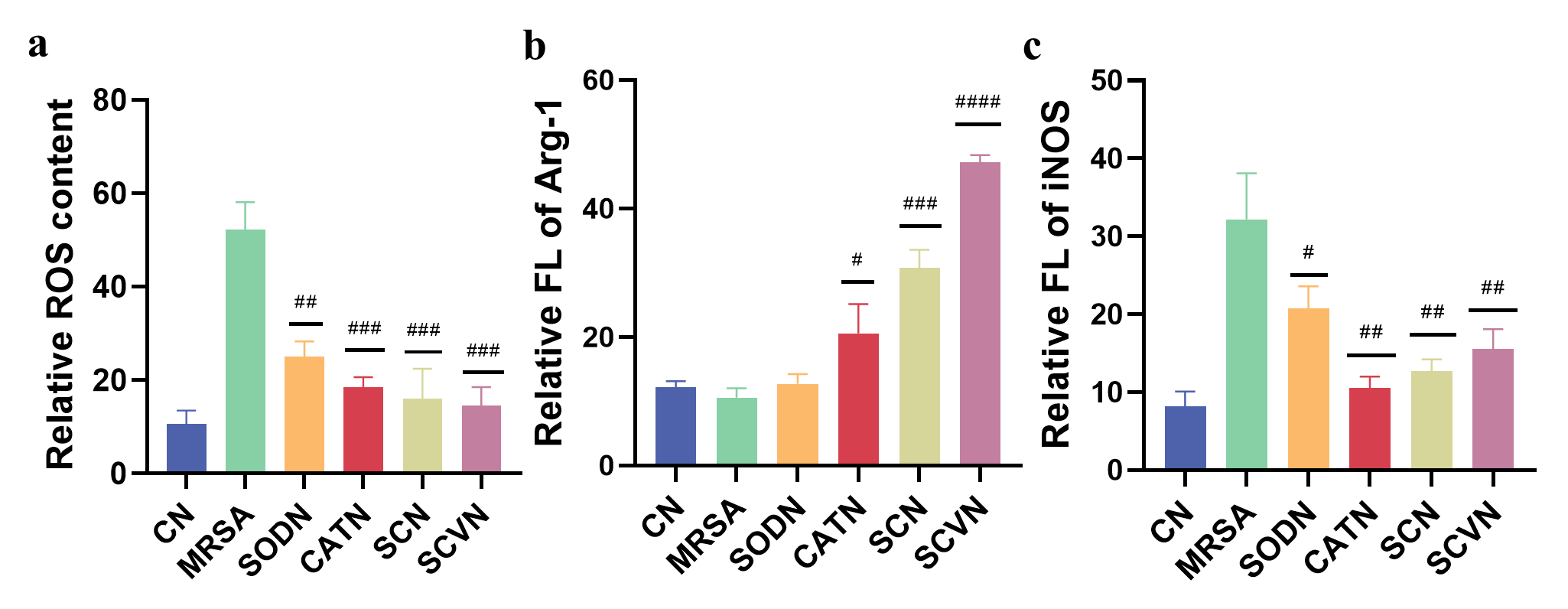


**Figure S23** Quantitative analysis of a) ROS level, b) Arg-1 and c) iNOS expression level in Figures 5i, 5j and 5k, respectively (n=3).


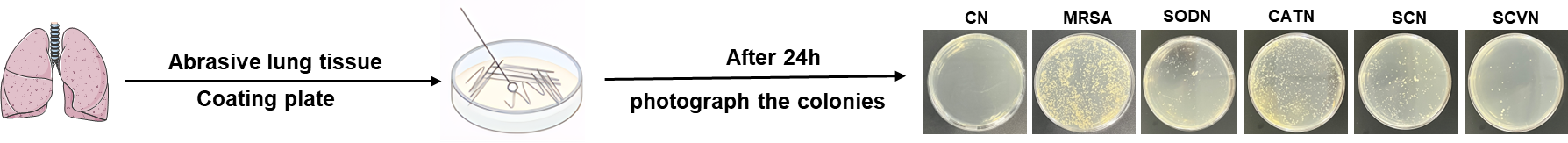


**Figure S24** Photographs of solid LB agar plates of bacterial colonization in lung tissues.


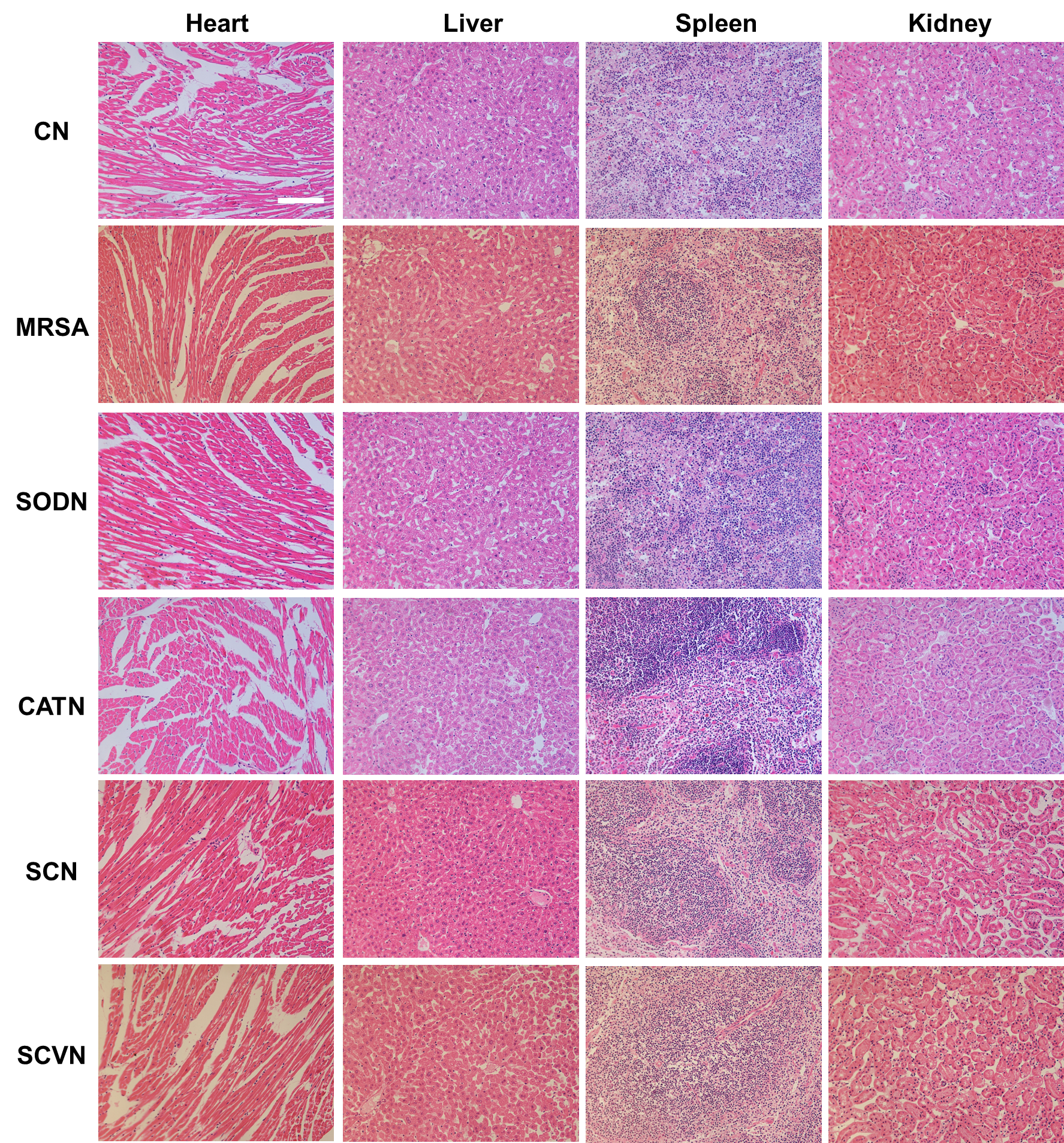


**Figure S25** H&E staining of heart, liver, spleen, kidney after therapeutic treatments. Scale bar: 50 μm.
